# Gastroparesis Phenotyping: Current Methods and Emerging Opportunities With Magnetic Resonance Imaging

**DOI:** 10.1111/nmo.70454

**Published:** 2026-10-07

**Authors:** Xiaokai Wang, Michael Camilleri, Zhongming Liu

**Affiliations:** ^1^ Department of Biomedical Engineering University of Michigan Ann Arbor Michigan USA; ^2^ Division of Gastroenterology and Hepatology Mayo Clinic Rochester Michigan USA; ^3^ Department of Electrical Engineering Computer Science University of Michigan Ann Arbor Michigan USA

**Keywords:** disease phenotyping, functional mapping, gastric emptying, gastrointestinal motility, gastroparesis, magnetic resonance imaging, precision medicine

## Abstract

**Background:**

Gastroparesis is a complex gastrointestinal motility disorder characterized by delayed gastric emptying. Its pathophysiology varies across patients, arising from impaired fundic tone, antral hypomotility, pylorospasm, antropyloroduodenal incoordination, or combinations thereof. Current clinical diagnostic tools provide only a fragmented assessment of gastric function and lack the comprehensive spatiotemporal details necessary to differentiate these pathophysiological phenotypes, limiting the ability to deliver personalized treatments.

**Purpose:**

To overcome this gap, we advocate for dynamic magnetic resonance imaging (MRI) as a non‐invasive “all‐in‐one” diagnostic platform for simultaneous evaluation of gastric accommodation, regional wall kinematics, pyloric function, and overall emptying within a single exam following the oral ingestion of a natural‐ingredient test meal. This integrated assessment offers a unique capability for gastroparesis phenotyping by characterizing how various types of regional motor dysfunction lead collectively to delayed gastric emptying in each patient. Quantifying MRI‐derived phenotypes using a standardized surface scaffold of the gastric musculature creates a functional mapping framework to contrast patient‐specific deviations against normative baselines established from healthy cohorts. The framework enables the integration of localized motor phenotypes with underlying structural knowledge for objective explanation of clinical symptoms and evidence‐based, personalized treatments. Ultimately, establishing dynamic MRI as the emerging diagnostic standard promises to transform gastroparesis management from an empirical practice into a new era of precision gastroenterology.

**Summary:**

Gastroparesis is a disease condition characterized by delayed gastric emptying, which may arise from different underlying causes that existing tests cannot fully distinguish. We review recent advances in dynamic MRI of the upper gastrointestinal tract and demonstrate that dynamic MRI can simultaneously assess gastric emptying, fundic tone, antral and duodenal contractions, and pyloric opening, all in a single examination. We highlight its potential to identify patient‐specific patterns of dysfunction and support more precise diagnosis and treatment.

## Key Points

- Gastroparesis may arise from distinct regional motor abnormalities, including impaired fundic tone, antral hypomotility, pyloric dysfunction, and antropyloroduodenal incoordination.
- Current diagnostic tests provide fragmented assessments and cannot fully characterize how these abnormalities interact to delay gastric emptying.
- Dynamic MRI provides an “all‐in‐one” platform to enable integrated, noninvasive assessment of gastric accommodation, wall kinematics, pyloric function, and emptying after a natural test meal.
- Standardized surface mapping of MRI‐derived motor function may support patient‐specific phenotyping and more targeted treatment.
- Further development, standardization, validation, and evaluation are needed for broad clinical adoption.

## Graphical abstract

Anatomical and dynamic MRI performed before and after ingestion of a natural test meal provide complementary assessments of gastric accommodation, emptying, and regional wall kinematics. This integrated, noninvasive examination supports mechanistic phenotyping of gastric motor dysfunction in gastroparesis.

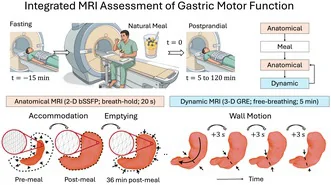

## 1 Introduction

The stomach's function in digestion requires the precise coordination of gastric accommodation, fundic tone, antral contraction, pyloric opening, and duodenal propulsion to effectively store, triturate, and propel food [1]. Pathophysiological changes in any individual or combination of these regional motor functions can lead to gastroparesis [2], characterized by delayed gastric emptying without mechanical obstruction [3, 4, 5]. Most commonly arising from idiopathic, diabetic, or post‐surgical etiologies [6], gastroparesis can cause chronic symptoms such as nausea, vomiting, abdominal pain, early satiety, and weight loss [4, 5], representing a major burden to patients and healthcare [7, 8, 9].

Clinical diagnosis of gastroparesis currently relies on endoscopy to rule out mechanical obstruction, followed by gastric emptying scintigraphy (or breath testing) to confirm delayed emptying [4, 10, 11]. These standard tests lack the spatiotemporal details necessary to differentiate how the different gastrointestinal regions are involved in the underlying pathophysiology. In fact, delayed gastric emptying can arise from distinct motor phenotypes that vary across patients and require personalized treatments [2, 5]. Typical phenotypes include impaired fundic tone [12], antral hypomotility [13, 14], pylorospasm [15, 16, 17], and antropyloroduodenal incoordination [18, 19, 20]. Each phenotype reflects a specific deficit in the intrinsic or extrinsic mechanisms of motility control [1, 2, 21, 22]. Therapeutics must target the pathophysiology in a patient‐specific manner to effectively restore motor function, normalize emptying, and relieve symptoms. However, for decades, clinical management of gastroparesis has been largely heuristic with suboptimal or even disappointing patient outcomes [4]. The field lacks a widely available diagnostic framework capable of granular and integrated assessment of gastric motor functions. This gap limits the ability to phenotype gastroparesis and to further deliver targeted treatments.

To fill this gap, we advocate for dynamic magnetic resonance imaging (MRI) as a noninvasive diagnostic modality for gastroparesis and other upper GI motility disorders [23, 24, 25, 26, 27]. Unlike existing methods, dynamic MRI offers high spatiotemporal resolution to simultaneously evaluate gastric accommodation, regional gastric motility, antropyloroduodenal coordination, and overall gastric emptying in a single exam, supported by findings from both animal and human MRI studies [23, 25, 26, 28, 29, 30, 31, 32, 33, 34, 35]. This integrated capability brings an emerging opportunity to characterize how regional motor functions contribute to normal or impaired gastric emptying. To elaborate on this perspective, we first summarize the distinct motor phenotypes of normal vs. delayed gastric emptying, existing diagnostic tools, then review recent work on dynamic MRI, and lastly outline a roadmap to establish dynamic MRI as a new diagnostic standard for precision medicine in gastroparesis.

## 2 Regional Motor Function and Neuromuscular Control

The stomach consists of structurally distinct regions that execute different motor functions [36]. The proximal stomach, including the fundus and proximal corpus, acts as both a reservoir and a pressure pump. Its smooth muscles relax to accommodate the incoming food during ingestion and maintain tonic contractions to propel ingesta towards the distal stomach during digestion. In turn, the distal stomach, including primarily the distal corpus and antrum, acts as a peristaltic pump. Its smooth muscles generate peristaltic contractions that oscillate in time at ~3 cycles per minute (cpm) and travel in space at ~3 mm per second in humans [26]. When the contractile wave reaches the distal antrum, the pyloric sphincter either contracts to cause shearing forces for food trituration or relaxes to allow passage of chyme into the duodenum. Upon the pyloric opening, the duodenum relaxes to accommodate the chyme and then contracts to propel the chyme further downstream. When it is overloaded, the duodenum delivers neurohormonal feedback to slow down gastric emptying [37, 38]. The precise and adaptive coordination of these motor processes ensures a proper rate of gastric emptying that depends on the meal's physical properties (volume, consistency, viscosity, and osmolarity) and nutrient contents (caloric density, carbohydrates, fats, and sugars) [1, 13, 34].

Gastric motor functions are governed by neuromuscular circuits specialized for each region [21, 36, 39, 40, 41]. The gastric musculature consists of smooth muscle cells (SMC) organized into longitudinal, circular, and oblique muscle layers [42]. Myenteric ganglia lie between these muscle layers, where enteric motor neurons provide excitatory or inhibitory inputs that regulate muscle contractions or relaxations, respectively [22, 43]. Neuromuscular transmission occurs through an electrically coupled syncytium comprising SMC, intramuscular interstitial cells of Cajal (ICC‐IM), platelet‐derived growth factor receptor‐α (PDGFRα) positive cells, collectively known as the SIP syncytium [44, 45, 46, 47, 48]. This intrinsic neural circuitry regulates tonic contractions and relaxations in the proximal stomach; in the distal stomach, it modulates phasic contractions paced by electrical slow waves generated and propagated by myenteric interstitial cells of Cajal (ICC‐MY) [49, 50, 51].

In addition, vagal efferent neurons innervate the excitatory or inhibitory enteric motor neurons through parallel pathways that originate from different subnuclei within the dorsal motor nucleus of the vagus (DMNv) [52] and branch out to innervate different gastric regions [21]. Together, the vagal‐enteric circuits provide parallel, balanced, and region‐specific excitatory or inhibitory regulation to orchestrate muscle contractions or relaxations, respectively [21, 53]. To close the loop with the vagal motor control [54], vagal afferent neurons with cell bodies in the nodose ganglia transmit sensory feedback from the gut to the central nervous system and innervate distinct gastric regions and layers with specialized nerve endings [21, 55, 56]. In particular, vagal afferents detect gastric tension via intraganglionic laminar endings (IGLE) [57] and track muscle stretch or length via intramuscular arrays (IMA) [58, 59]. These mechanosensory signals give rise to specific responses at the nucleus tractus solitarius (NTS) [54, 60, 61, 62] and further activate higher regions for hierarchical interoceptive processing [63, 64, 65, 66, 67, 68]. Compared to the parasympathetic (vagal) innervation, the sympathetic (spinal) innervation of the stomach is not so important in the physiological regulation of gastric emptying [1, 40, 54, 65], although sympathetic adrenergic innervation is known to regulate pyloric tone [69].

## 3 Motor Phenotypes of Gastroparesis

Pathological damage at any level of this hierarchical motility control may disrupt the physiological process of normal gastric emptying. In gastroparesis, such disruption can delay gastric emptying through the various pathophysiological mechanisms that manifest one or multiple of the following motor phenotypes [2, 5].

### 3.1 Impaired Fundic Tone

During meal ingestion, the fundus relaxes to accommodate food; after ingestion, fundic tone helps transfer ingesta from the proximal reservoir towards the distal stomach. If fundic tone is impaired, the proximal stomach does not sustain an intragastric pressure gradient towards the distal stomach. Consequently, food stalls in the proximal reservoir and fails to reach the distal pump for active trituration and propulsion. Impaired fundic tone may arise from abnormal vagal‐enteric regulation of the fundic muscle either by increased inhibition that manifests as excessive gastric accommodation [12] or by decreased excitation that manifests as slower reduction of fundic volume in the early postprandial phase [70]. It is worth noting that abnormal fundic relaxation during meal ingestion is physiologically distinct from impaired postprandial fundic tone that contributes to delayed gastric emptying. Although impaired accommodation is not a defining feature of gastroparesis, accommodation abnormalities may co‐exist with delayed gastric emptying and likely contribute to the overlapping symptoms of gastroparesis and functional dyspepsia.

### 3.2 Antral Hypomotility

While the intragastric pressure gradient is sufficient for emptying of liquids [71, 72], the antrum must generate occlusive contractions to grind and triturate solids before they can pass through the pylorus [1, 13]. In certain pathophysiological conditions, antral contractions may become weaker, less frequent, or even absent, which are collectively termed antral hypomotility. These conditions can arise from damage to muscle cells, ICCs, or neuromuscular circuits in the distal stomach. Physiologically, the ICC‐MY operates as a network of coupled oscillators with spatially variable intrinsic frequencies [49, 50, 73]. The intrinsic frequency is highest at a pacemaker zone in the proximal stomach and decreases progressively towards the pylorus [50, 74]. Normally, the pacemaker entrains the entire ICC‐MY network such that electrical slow waves propagate distally to pace and synchronize phasic contractions at the same frequency throughout the antrum [75]. When the ICC‐MY network is depleted or compromised [76], this electrical entrainment breaks down [77, 78], causing the antrum to uncouple from the dominant pacemaker and revert to slower, irregular frequencies, namely gastric dysrhythmia [79, 80, 81]. Moreover, a loss of excitatory neural regulation impairs muscle contraction, and a loss of inhibitory neural regulation impairs muscle relaxation [2]. Together, these deficits restrict the dynamic range of circumferential wall motions such that the antrum may fail to drive the axial and shearing forces necessary to triturate and propel food [82, 83, 84].

### 3.3 Pylorospasm

Excitatory neuromuscular circuits allow the pyloric sphincter to generate both tonic and phasic contractions for the grinding, mixing, and trituration of digestible solids into small particles and liquefied chyme. Conversely, inhibitory neuromuscular circuits control the relaxation of the pyloric sphincter to open the pylorus for the chyme to pass through. When this inhibitory control is compromised, the pyloric sphincter remains in a state of persistent tonic contraction. This condition, known as pylorospasm, increases pyloric resistance and impedes gastric outflow despite active propulsion by antral contractions [15, 16, 17]. Phenotypically, pylorospasm manifests as an elevated pyloric tone, infrequent or inadequate pyloric opening.

### 3.4 Antropyloroduodenal Incoordination

Effective gastric emptying relies on precise spatiotemporal coordination across the antrum (A), pylorus (P), and duodenum (D), which form a highly integrated, adaptive antropyloroduodenal (APD) motor complex. The APD coordination is a triad of two‐way interactions, including antropyloric coordination, pyloroduodenal coordination, and duodenogastric feedback. This coordination depends largely on neural and/or neurohormonal signaling. Gastric and duodenal contractions do not share a common pace‐making activity, as the gastric and duodenal ICC networks are not continuous across the pyloric sphincter and their slow waves occur at notably different frequencies. Disruption of these interactions may delay gastric emptying (characteristic of gastroparesis) or accelerate it (characteristic of dumping syndrome) [85].

First, antropyloric coordination is essential for both the transpyloric transit of chyme and the trituration of solids. Upon peristaltic arrival at the terminal antrum, an open pylorus allows chyme passage, whereas a closed pylorus causes retropulsion to grind digestible solids [86]. Disruption of this coordinated transition delays gastric emptying, either because incomplete grinding leaves solids too large to pass or excessive retropulsion impedes anterograde transit.

Second, upon transpyloric transit, the duodenum must first relax to accommodate the incoming chyme before generating rhythmic segmentation to mix the chyme with biliary and pancreatic secretions, followed by peristaltic contractions to further propel the chyme downstream [87]. However, when such structured duodenal contractions become uncoordinated, they can create excessive retrograde pressure gradients that abnormally oppose gastric outflow, causing chyme to stall or even reflux into the stomach [88].

Lastly, duodenogastric feedback provides a brake mechanism to prevent intestinal overload [38]. Through the release of gut peptides, e.g., cholecystokinin (CCK) [89], glucagon‐like peptide‐1 (GLP‐1) [90], and peptide YY (PYY) [91], neurohormonal signaling from the duodenum back to the stomach regulates antral motility and pyloric tone based on nutrient contents and caloric load [37, 92]. When this feedback loop is overactive, aberrant inhibitory signaling can prematurely suppress antral motility or close the pylorus, while slowing down transit through the duodenum.

## 4 Non‐MRI Testing of Gastric Emptying and Motility

Current diagnostic testing of gastroparesis relies primarily on methods that separately assess gastric emptying, accommodation, or antropyloroduodenal motility. Next, we review existing non‐MRI methods, their advantages and limitations, before discussing MRI in the sections that follow.

The clinical diagnosis of gastroparesis relies on documenting the overall delay in gastric emptying. The gold standards for evaluating gastric emptying are scintigraphy [93, 94] and stable isotope (^13^C‐spirulina) breath testing [95, 96], which have been cross‐validated in terms of the half‐emptying time during concomitant measurement of the same dually‐labeled meal [96]. Clinically, gastroparesis is confirmed by scintigraphic evidence of delayed emptying with gastric retention > 60% at 2 h or > 10% at 4 h following a standardized 250 kcal, 2% fat egg protein substitute solid test meal [93]. Alternatively, a 320 kcal, 30% fat meal can be used to document delayed emptying by > 75% retained at 2 h and > 25% retained at 4 h [94]. Although conventional gastric emptying scintigraphy only reports overall emptying parameters, dynamic antral scintigraphy [97] and its implementation with recent Fourier phase‐analysis software [98] can also characterize antral contraction. However, scintigraphy is a planar (2‐D) imaging technique with limited spatial resolution, and it cannot directly resolve 3‐D structures or their dynamics necessary to assess continuous wall motions across the stomach, pylorus, and duodenum.

Accommodation and emptying are different phases of gastric function but share the same underlying vagal‐enteric‐muscular circuits. Like two sides of the same coin, the pathophysiological defects in the proximal stomach that can delay emptying may also leave a related signature on accommodation. To evaluate gastric accommodation, the gold standard is the gastric barostat, which measures volume changes via a constant‐pressure intragastric balloon [99, 100]. However, it is not commonly used in clinical practice because its mechanical perturbation alters postprandial physiology [101, 102]. Noninvasive alternatives also have limitations. Single‐photon emission computed tomography (SPECT) uses intravenous radioactive technetium‐99 m (^99m^Tc) to label the gastric mucosa and estimate the gastric volume [103, 104]; but it requires ionizing radiation and cannot differentiate ingested food from gastric secretions or air. Ultrasonography [105, 106, 107] is compromised by operator dependency, acoustic shadowing from intraluminal gas, and restricted access to the stomach under the left ribs.

Effective emptying requires active trituration, propulsion, and mixing of digestible solids by the antrum, pylorus, and duodenum. To evaluate gastroduodenal motility, the gold standard is the high‐resolution manometry [108], which uses a transnasal or transoral catheter to record intraluminal pressure with water‐perfused or solid‐state sensors placed along the antropyloroduodenal junction [14, 109]. Manometric data report antral motility, pyloric function, and duodenal motility during either normal or impaired emptying [13, 14, 18, 19, 109, 110, 111, 112, 113]. However, the procedure requires intubation and specialized expertise limited to few tertiary centers [14, 109, 111, 114]. Despite the use of tens of sensors, intraluminal pressure is only sampled at discrete locations and thus is unable to capture the continuous pattern of muscle contraction. Alternatively, wireless motility capsules, such as the legacy SmartPill [115, 116, 117] and the newer Atmo [118, 119], are ingestible devices to record luminal parameters (e.g., temperature, gas, and acceleration) during gastrointestinal transit. As a rigid, free‐floating device, the capsule lacks accurate spatial information and does not reflect the actual transit of chyme.

Lastly, body surface gastric mapping (BSGM) uses a cutaneous electrode array to capture gastric slow‐wave patterns [120, 121]. The recent “Auckland Classification” provides consensus definitions and standardized criteria for six BSGM phenotypes based on myoelectrical features or symptom patterns [122]. Although BSGM is noninvasive and reproducible, it does not directly measure regional contractions, pyloric function, gastric emptying, or their interactions in gastroparesis [123]. Biophysical and physiological interpretation is also complicated by volume conduction, which limits localization and differentiation of many electrical sources contributing to body‐surface potentials. Moreover, consensus‐based classification does not establish that each phenotype reflects a specific and distinct pathophysiological mechanism.

A critical limiting segment for gastric emptying is the pyloric sphincter. High‐resolution manometry can capture both tonic and phasic contractions of the pyloric sphincter. Elevated pyloric tone uncoordinated with antral contractions indicates pylorospasm [14]. In addition, the endoluminal functional lumen imaging probe (EndoFLIP) uses controlled mechanical distension to probe the distensibility of the pylorus (i.e., pyloric compliance) [124, 125]. However, this procedure is invasive, requires sedation, and does not necessarily replicate the physiology associated with dynamic chyme transit.

Each of the diagnostic methods described above provides only a fragmented assessment of gastric emptying, accommodation, or motility [126]. For a comprehensive evaluation, a patient must undergo separate exams to assess distinct aspects of gastric function: for example, gastric emptying with scintigraphy, accommodation with a gastric barostat or SPECT, and antropyloroduodenal motility with high‐resolution manometry. Without simultaneous acquisition, separate modalities cannot be combined to report the integrated process by which distinct types of motor events interact or interfere with one another to give rise to normal or impaired gastric emptying. It is impractical to link data across exams and modalities due to within‐individual variation and the highly distinct time scales across different modalities.

## 5 Dynamic MRI for “All‐In‐One” Evaluation

MRI is a promising yet under‐utilized method to assess gastric motor function [127, 128]. As shown in Figure 1, dynamic MRI can simultaneously evaluate gastric emptying, regional volumes, wall motion, and transpyloric transit, potentially surpassing or replacing existing diagnostic standards. The integrated MRI assessment can be accomplished in a single exam without any invasive procedures, ionizing radiation, synthetic contrast agents, or non‐physiological manipulations. This premise rests upon recent progress, including natural ingredients for MRI‐visible test meals, accelerated MRI acquisition, and automated MRI analysis (see details in Appendix 1). Below, we discuss strategies for using dynamic MRI to evaluate the motor functions of the stomach, focusing on recent progress beyond earlier reviews [129, 130] while excluding MRI applications to the intestines reviewed elsewhere [127, 128, 131].

**FIGURE 1:**
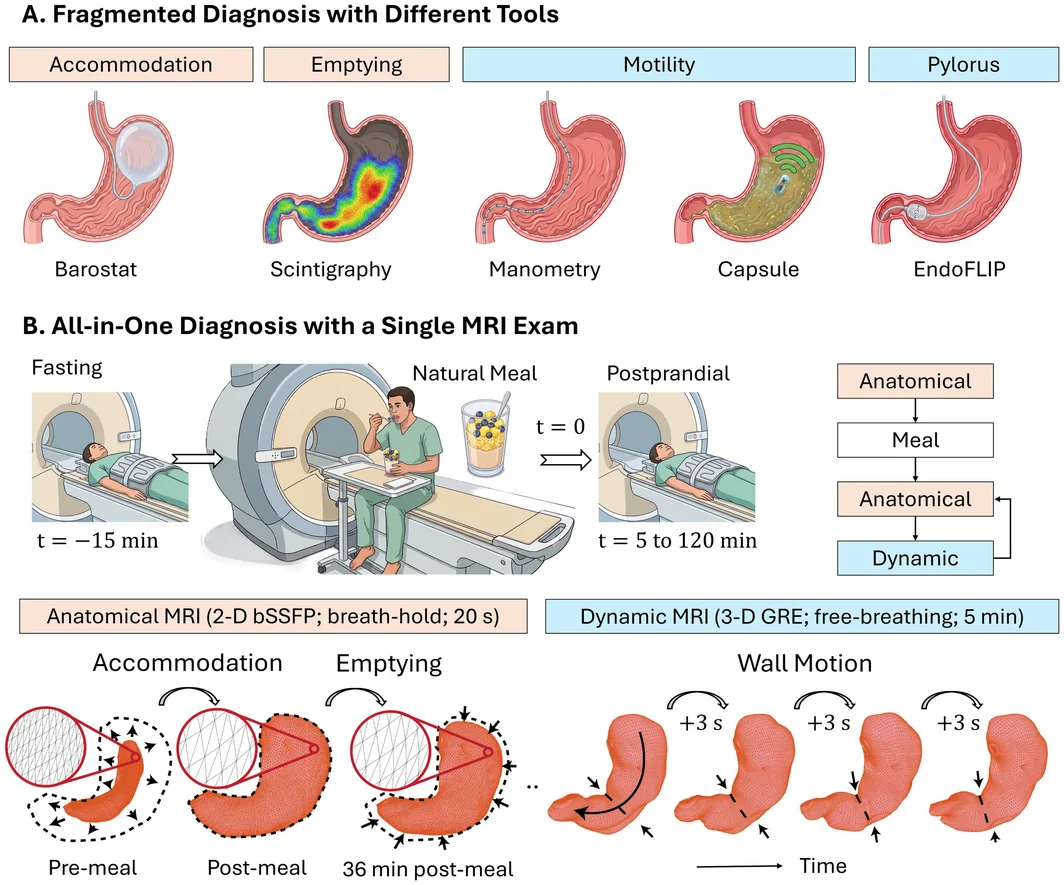
Paradigm shift from fragmented clinical testing to noninvasive, integrated MRI assessment of gastric motor function. (A) Existing methods for testing specific aspects of gastric motor function, such as the gastric barostat for accommodation, scintigraphy for emptying, high‐resolution manometry and wireless motility capsule for regional motility, and EndoFLIP for pyloric distensibility. (B) Clinical workflow for using MRI to evaluate multi‐faceted gastric functions within a single exam. The MRI protocol requires a human subject to fast overnight. The next morning, the subject first receives MRI in the fasting condition, then consumes a standardized natural test meal (with the completion time defined as t=0), and continues MRI for 1 or 2 h. Postprandial MRI sessions alternate between anatomical MRI (2‐D bSSFP with a 20‐s breath‐hold) and dynamic MRI (3‐D GRE with free‐breathing for 5 min). Anatomical MRI evaluates the gastric volume, morphology, and their changes over time. The changes from pre‐meal to post‐meal quantify gastric accommodation. Subsequent changes characterize gastric emptying. Dynamic MRI reveals gastric wall kinematics with temporal resolution of 2–3 s.

### 5.1 Natural Test Meal

MRI‐based assessment of postprandial gastric function follows the oral ingestion of a test meal. Earlier studies used liquid meals doped with gadolinium to enhance contrast [132, 133, 134, 135, 136, 137, 138, 139, 140, 141]. However, liquids and solids engage fundamentally different processes during gastric emptying [1, 13]. Liquid meals are suboptimal for evaluating gastroparesis [4, 5, 94]. Using gadolinium also poses safety concerns [142].

Instead, recent studies use natural ingredients to formulate a solid or semi‐solid test meal and demonstrate robust contrast for dynamic MRI [25, 26, 27]. One formulation blends pineapple, blueberry, banana, and firm tofu into a 350 g, 236 kcal test meal with a balanced nutrient profile (74% carbohydrate, 7% fat, 13% protein, and 6% fiber) [26, 27]. Another formulation blends 400 g of pineapple (200 kcal) into 150 g of instant vanilla pudding to create a lactose‐free, 560 kcal, 470 mL semi‐solid meal [25]. Both formulations include pineapple for its naturally high manganese (Mn^2+^) content [143]. By shortening the longitudinal relaxation time (T_1_), Mn^2+^ ions enhance contrast in T_1_‐weighted MRI and enable rapid acquisition [62, 144]. Furthermore, the natural meal can be adjusted in viscosity and nutrients for specific clinical needs, or incorporate standard ingredients (e.g., eggs and Ensure) used for gastric scintigraphy [94].

### 5.2 Gastric Accommodation

MRI evaluates gastric accommodation by quantifying the postprandial increase in gastric volume compared to a fasting baseline. Accurate evaluation requires anatomical scans of the entire stomach acquired during ~20‐s breath‐holds before and immediately after a test meal (Figure 1B) [26, 27, 135, 145]. With high resolution and contrast, these images can delineate the gastric wall for 3‐D stomach reconstruction and differentiate intragastric contents, including the meal, air, and secretions (Figure 2, top) [23, 134, 135, 137]. Applying a deformable surface model to the 3‐D lumen captures the surface expansion of the stomach (Figure 1B) [26], attributing volumetric accommodation to local muscle stretch [26, 28]. This mapping is essential because normal or impaired accommodation is driven by gastric wall biomechanics, of which intraluminal changes are the observable consequence. Furthermore, MRI measurements of gastric accommodation have been cross‐validated with SPECT [135] and the gastric barostat [146]. Since MRI offers superior resolution and contrast without invasive mechanical perturbation by the intragastric barostat‐controlled balloon, it is expected to provide a more accurate and granular assessment of truly physiological accommodation [102, 135, 147].

**FIGURE 2:**
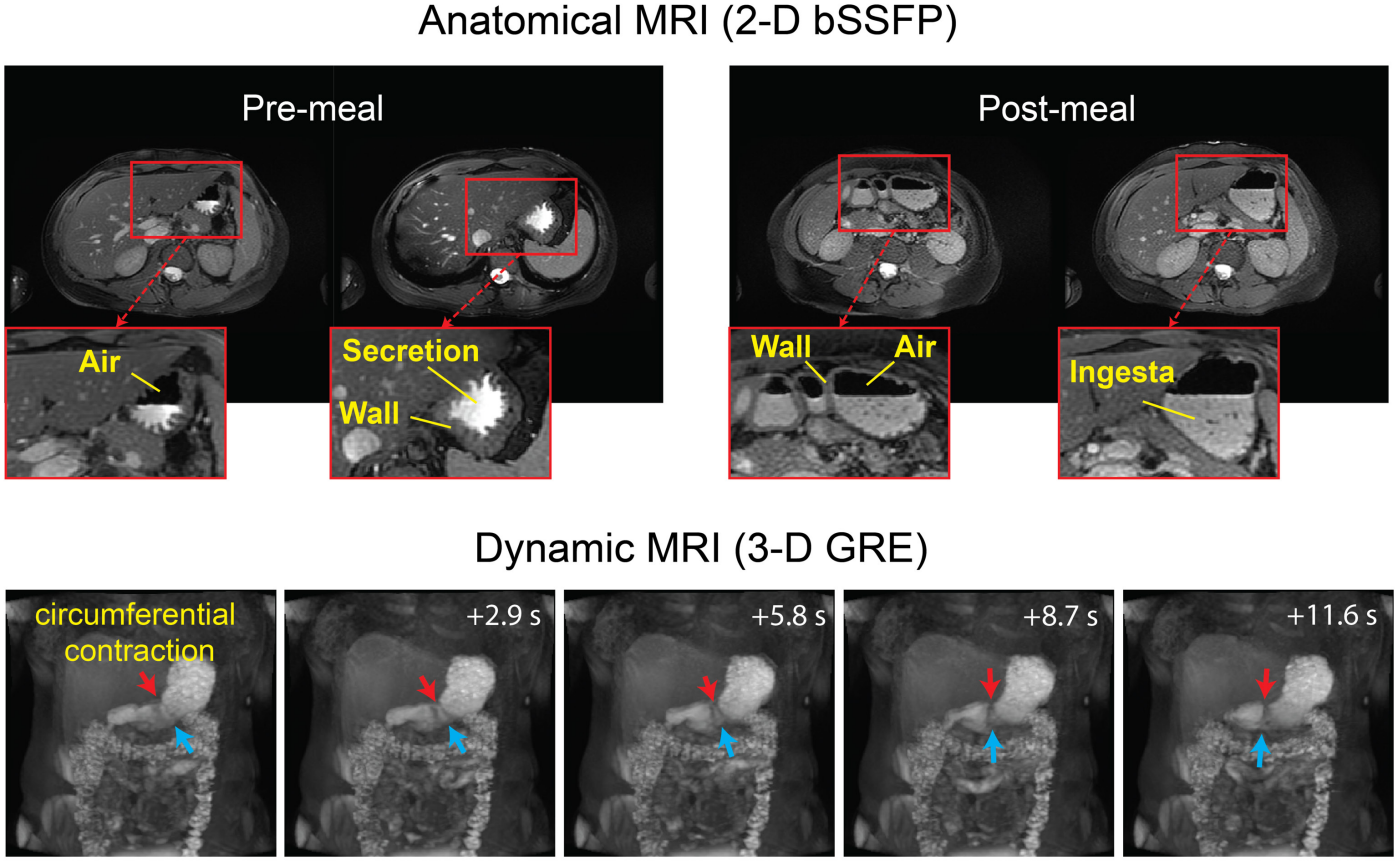
Anatomical and dynamic MRI. (Top) Anatomical MRI differentiates the gastric wall, luminal air, and secretions pre‐meal vs. post‐meal for quantitative assessments of accommodation and emptying. (Bottom) Dynamic MRI captures real‐time gastric motility. The time‐series shows a distally propagating circumferential contraction (arrows) traversing the stomach over a 12 s window. The images shown are the maximal intensity projections of 3‐D MRI at each time frame.

### 5.3 Gastric Emptying

Similarly, repeated anatomical MRI can evaluate gastric emptying [23, 26, 34, 132, 135, 136, 137, 138, 148, 149, 150]. A sequence of breath‐hold MRI scans acquired every 5 to 15 min after the test meal can track the postprandial changes in the gastric volume [132, 148] and its partition into distinct regions (fundus, corpus, antrum) and contents (food, air, secretions). Fitting a parametric (linear or exponential) model to the time‐varying volume estimates key parameters that characterize the emptying kinetics, such as the half‐emptying time, the fractional gastric retention at 1, 2, or 4 h. These MRI‐derived parameters correlate with gastric emptying scintigraphy for both liquids [132, 135] and solids [23, 26, 148, 149], depend on meal viscosity and nutrient composition [34, 136], and report characteristic distinctions among normal, delayed, and accelerated emptying in health and disease or in response to pharmacological interventions [132, 133, 151, 152].

### 5.4 Gastric Motility

With sufficiently accelerated acquisition, dynamic MRI can characterize gastric motility or dysmotility (Figure 2, bottom) [25, 26, 27, 35, 131, 138, 141, 153, 154, 155]. Recordings from the human stomach suggest that normal peristaltic contractions exhibit a fundamental frequency of approximately 3 cpm (or 0.05 Hz) within a physiological range of 2.5–3.75 cpm, whereas dysrhythmia manifests as bradygastria (0.5–2.5 cpm) or tachygastria (3.75–10 cpm) [81]. To characterize gastric motility without temporal aliasing, dynamic MRI must scan the entire stomach in < 8 s to cover the normal range or < 3 s to cover the full range. Satisfying these criteria is technically demanding, because the postprandial stomach requires a large 3‐D field of view (typically 360×360×140 mm^3^ depending on the body size), while the postprandial thickness of the gastric wall (2 to 5 mm) requires high spatial resolution (ideally < 2 mm). When these criteria are met, integrating volumetric (3‐D) and dynamic (1‐D) data yields 4‐D information about gastric motility (Figure 3), hereafter referred to as 4‐D MRI.

**FIGURE 3:**
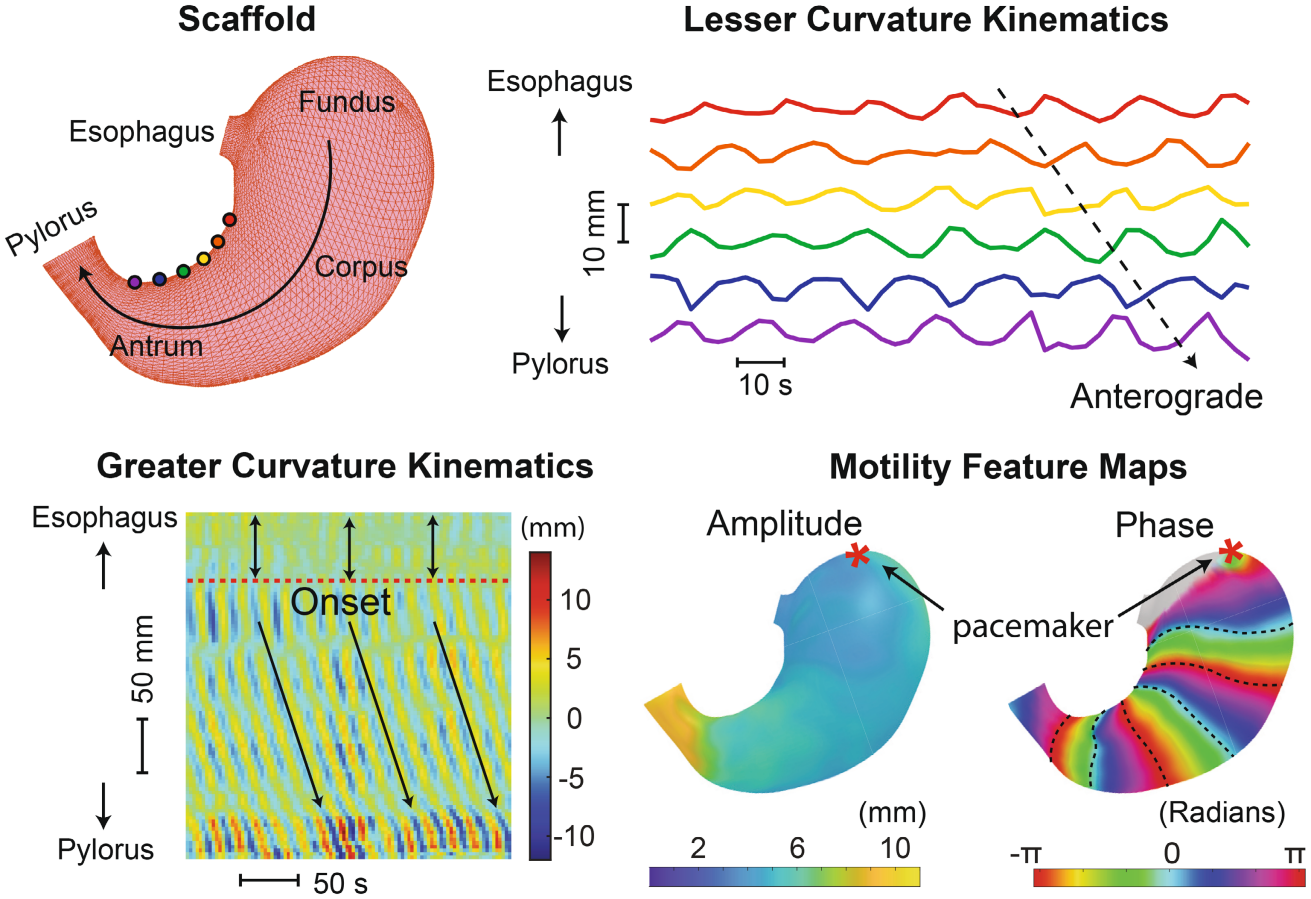
Surface mapping of gastric motility. (Top) Diffeomorphic transformation of a standardized scaffold of the gastric musculature fits dynamic MRI data and captures biomechanical events. For each location on the scaffold, the kinematic time‐series reflects underlying muscle contractions and relaxations. The temporal shifts across locations reflect the propagation of the contractile waves, e.g., anterograde propagation from the proximal to distal antrum as illustrated with representative points along the lesser curvature. (Bottom) Space–time plot of wall motions along the greater curvature. Negative and positive values indicate inward and outward movements. Red dashed lines mark the peristalsis onset that initiates the distal traveling waves (unidirectional arrows). The amplitude and phase are mapped onto the stomach template. The amplitude map highlights progressively increased contraction strength in the distal antrum. The phase map shows the spatially organized peristaltic contractions.

Dynamic MRI protocols should balance spatial coverage with spatiotemporal resolution. While single‐slice 2‐D acquisitions offer high spatial (1.5–2.5 mm) and temporal (< 2 s) resolution [35, 138, 147, 153, 156], the stomach's complex 3‐D morphology prevents any single plane from capturing non‐uniform contractions. Extension to a few parallel slices remains insufficient [141, 147, 155] due to out‐of‐plane movements. In contrast, state‐of‐the‐art protocols ensure sufficient coverage while pushing the limits of spatiotemporal resolution using 3‐D MRI acquisition with parallel imaging [25, 26, 27]. Comparing their native (without any interpolation) equivalent isotropic resolutions, recent studies achieved 2.5mm spatial resolution at 7s temporal resolution [25], 2 mm at 3.3 s [27], and 2.7 mm at 3 s [26]. Temporal resolution should be near or below 3 s to prevent temporal aliasing, which can distort the measured motility. A 7s sampling rate is borderline for normal contractions and inadequate for faster events or rhythms.

Increasing evidence supports the validity and sensitivity of dynamic MRI for evaluating gastric motility. Studies with simultaneous dynamic MRI and manometry [71, 157, 158] show that ~80% of MRI‐observed contractions are also detected by manometry [158], with wall motion correlating highly with intraluminal pressure during both active and inactive periods of antral contractions [157]. Similarly, concurrent MRI and intragastric barostat recordings demonstrate consistent “volume waves” indicative of global rhythmic wall motions [147]. Dynamic MRI is sensitive to motility changes induced by vagus nerve stimulation in animals [31, 32] and in response to various pharmacological agents in humans, such as loxiglumide [138], erythromycin [147], metoclopramide [156], scopolamine [156], and glucagon [156].

From 4‐D MRI, it is feasible to infer the active tension and motion of the gastric wall that propels luminal contents [26, 27, 28]. Recent studies model gastric wall motions by applying differential geometry and neural networks to dynamic, volumetric MRI data [28]. As shown in Figure 3. this analysis tracks and quantifies continuous kinematics and creates functional maps on a standard surface template of the stomach [26, 28], providing a unified framework to report motility features (e.g., the amplitude, frequency, velocity, and coordination of peristaltic contractions) with high physiological fidelity [26].

### 5.5 Pyloric Function

As the gateway between the stomach and the duodenum, the pylorus has thicker muscle layers (up to 8 mm) and denser neural innervation than the antrum [69, 159]. While the pyloric canal is 2–3 cm long, the pyloric sphincter spans only 3–6 mm, serving as the “choke point” that controls transpyloric transit. As dynamic MRI relies primarily on ingested luminal contrast, the pylorus appears dark when closed and only appears bright during its brief opening phases. To resolve the pylorus, 3‐D MRI acquisitions should use higher in‐plane resolution (< 2 mm) within slices parallel to the long axis of the stomach, and as a tradeoff, use lower resolution in the slice‐encoding direction. While 2‐D acquisitions are likely to miss the pylorus entirely, 4‐D MRI can localize the pylorus based on geometric and temporal context. Specifically, the lumen progressively narrows as it approaches the pylorus from the antrum and abruptly expands as it enters the duodenal bulb.

Dynamic MRI assessment of pyloric function has been rarely reported. Preclinical studies in rats demonstrate the feasibility of using MRI to identify the pylorus and track its opening and closing [31, 32]. Their results show that intermittent pyloric openings normally occur at a similar frequency as antral contractions, and that vagus nerve stimulation can enlarge and prolong the pyloric opening to accelerate gastric emptying [32]. Recent efforts extend this feasibility by demonstrating MRI‐observed pyloric function in both healthy controls and patients with gastroparesis [160, 161]. Nevertheless, the pylorus remains a challenging target for MRI, requiring ongoing technical development to further improve spatiotemporal resolution for human applications.

### 5.6 Duodenal Motility

The duodenum accommodates gastric outflow through receptive relaxation and generates complex patterns of phasic contractions to mix and propel chyme [87]. These contractions are paced by an intrinsic electrical rhythm at ~12 cpm (or 0.2 Hz), which manifests as sustained contractile activity during Phase III of the interdigestive migrating motor complex [162]. Postprandially, duodenal contractions become intermittent as enteric neurons regulate excitability in response to episodically delivered chyme. Controlled by the frequency (~3 cpm) and synchrony of antral contractions and pyloric opening, each delivered bolus triggers a brief burst of contractions separated by quiescent periods [92]. While the average contraction frequency is significantly reduced in motility disorders [163], individual contractions within a burst remain phase‐locked to the 12‐cpm intrinsic pacemaker rhythm [164]. To fully capture such rapid, complex duodenal kinematics, 4D MRI should achieve a high temporal resolution of < 2.5 s (ideally < 1.5 s) alongside a high spatial resolution of < 2 mm. Because current MRI protocols hardly satisfy these specifications, reliable mapping of postprandial duodenal motility using 4‐D MRI remains a challenge.

### 5.7 Intraluminal Flow

Dynamic MRI captures gastric wall motions driving intraluminal flow and transit. Tracking voxel patterns estimates “optical flow” [165], while nonlinear registration infers displacement fields [166, 167]. Though these computational surrogates require validation, tracking MRI‐visible ingestible capsules offers a direct alternative [168]. Additionally, phase‐contrast MRI encodes proton velocity into the signal phase [169]. While established for cardiovascular MRI [170], it has been rarely applied to the stomach due to its significantly lower flow velocity, despite initial demonstration of feasibility [169]. Moreover, radiofrequency tagging superimposes a trackable grid onto luminal contents to infer flow [171, 172]; however, progressive tag fading from longitudinal (T_1_) relaxation limits this strategy, particularly with meals that shorten T_1_.

### 5.8 Cross‐Species Translation

As a noninvasive, high‐resolution imaging platform, dynamic MRI is applicable to both animals and humans and is thus well suited for cross‐species comparisons of gastric motor function. Comparative MRI studies report similar patterns of antral peristalsis in rats and humans, although at species‐specific frequencies of 5 and 3 cpm, respectively [26]. Notable differences occur in the proximal stomach. In rats, phasic contractions are largely confined to the distal stomach, whereas in humans they extend more broadly, appearing as non‐propagating “standing waves” in the fundus before transitioning distally into traveling waves at the distal fundus or proximal corpus [26]. This transition region defines the mechanical onset of propagating peristalsis, but does not necessarily indicate the electrical pacemaker. These MRI findings are consistent with intracellular electrophysiological and isometric recordings from resected human gastric muscles showing slow waves accompanied by phasic contractions in the fundus, as well as corpus and antrum [173]. However, whether the non‐propagating, phasic contractions in the fundus contribute to the fundic tone and whether they are altered in gastroparesis or functional dyspepsia remain to be investigated.

## 6 Emerging Opportunities

Dynamic MRI enables the granular and integrated evaluation of multi‐faceted gastric motor functions, replacing multiple separate diagnostic procedures with a single, noninvasive exam (Figure 1). MRI measures gastric volume and morphology with greater reliability than ultrasound and SPECT, evaluates gastric accommodation without the intragastric balloon, reports gastric emptying without ionizing radiation, captures gastric motility beyond discrete sites of manometric recording, and quantifies 3‐D wall kinematics with greater specificity than dynamic scintigraphy or motility capsule. Synergistically, these “all‐in‐one” capabilities create emerging opportunities to phenotype region‐specific motor dysfunction and pathophysiology. Defining patient‐specific phenotypes can ultimately identify underlying mechanisms, explain clinical symptoms, and guide targeted treatments.

To realize this phenotyping, 4‐D MRI enables the mapping of distinct motor functions (e.g., accommodation, motility, and coordination) onto a standardized surface template (or scaffold) of the stomach [26]. Aggregating these maps from healthy controls establishes a normative physiological distribution. Statistically contrasting a patient's functional map against this distribution localizes and quantifies specific deviations, yielding a first‐level, patient‐specific motor phenotype. While this first‐level phenotyping identifies many types of dysfunctions in isolation, second‐level phenotyping evaluates how regional motor dysfunctions collectively lead to a single physiological endpoint: the delayed gastric emptying. To quantify this “many‐to‐one” relationship, an empirical regression model that uses regional motor features as regressors to predict the rate of gastric emptying can be fitted with MRI measurements to quantify the relative contribution of each regional motor function to the overall delay of emptying. Alternatively, a mechanistic approach utilizing computational fluid dynamics (CFD) can simulate gastric outflow with boundary conditions specified by MRI‐observed gastric morphology, wall kinematics, and pyloric dynamics [174, 175, 176, 177], thus explaining gastric emptying with a biophysically realistic “virtual stomach”. Whereas CFD is computationally demanding, the empirical regression model is much simpler yet still effective in differentiating the biomechanical components that drive gastric emptying. Figure 4 illustrates the workflow for MRI‐based gastroparesis phenotyping.

**FIGURE 4:**
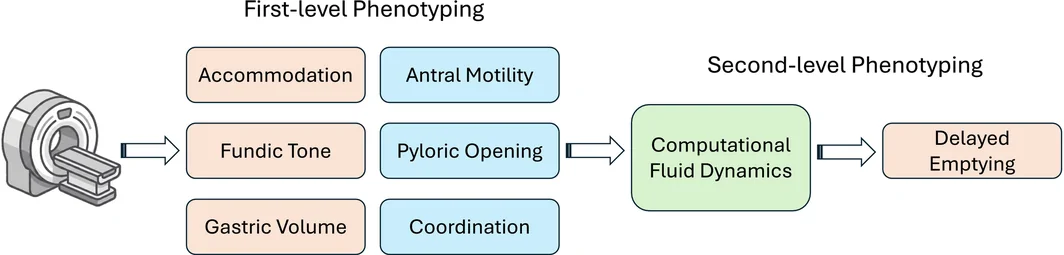
MRI phenotyping of gastroparesis. For a patient with symptomatic gastroparesis, a single MRI exam can derive first‐level phenotypes of gastric motor function, including accommodation, antral motility, and antropyloric coordination. A computational fluid dynamics model can further evaluate how regional motor dysfunctions contribute to delayed gastric emptying, deriving second‐level phenotypes.

Mapping MRI‐derived motor phenotypes to precise gastric locations provides a critical window into the cellular and circuit mechanisms underlying observed functional deficits. Each gastric region performs distinct motor functions through its specialized vagal‐enteric‐muscular circuitry [21, 40]. To bridge function with structure, the standardized scaffold is not only a template for functional evaluation [26], but also an atlas for the curation of structural data (e.g., immunohistochemistry, neuronal tracing, and histology) [178]. As such, this scaffold serves as a common coordinate system for registering the stomach's motor functions or phenotypes with its structural blueprint, defined by cell types, chemical codes, and circuitry patterns. Through this blueprint, knowing exactly “where” a motor function fails may offer structural clues as to “why.”

For example, MRI phenotypes of antral hypomotility localize pathophysiological changes in contraction frequency, amplitude, and coordination to specific structural circuits that control these features. Irregular frequency or uncoordinated propagation of antral contractions can be attributed to a focal loss of ICC or damage to the proximal pacemaker [2, 79, 80]. Weakened amplitude points to a focal loss of cholinergic enteric motor neurons or their intramuscular transducers [40]. Beyond these regional deficits, a phenotype of antropyloric incoordination isolates a breakdown in the extrinsic innervation, as vago‐vagal reflexes are essential to coordinating antral motility and pyloric opening [21, 54].

Furthermore, MRI‐derived motor phenotypes may explain gastrointestinal symptoms. The gastric wall contains distinct mechanoreceptors that sense abnormal stretch and tension to influence autonomic and central nervous systems through interoceptive signaling [57, 58, 59, 179], giving rise to patient‐specific clinical symptoms [180]. For example, impaired fundic accommodation rapidly increases proximal wall tension to trigger early satiety [99]. Both antral hypomotility and pylorospasm cause postprandial fullness and nausea [16, 79, 181].

Defining localized motor phenotypes shifts clinical management from “trial‐and‐error” to precision therapeutics [4]. While conventional approaches often prescribe treatments empirically, MRI‐derived phenotypes can inform targeted interventions. Specifically, impaired fundic accommodation rationalizes the use of fundic relaxants [182]. Antral hypomotility motivates the use of prokinetic agents [10]. Localized elevation of pyloric tone identifies candidates for pyloric sphincter interventions, such as botulinum toxin injection [183] or gastric peroral endoscopic myotomy (G‐POEM) [184, 185].

Dynamic MRI may also facilitate preclinical‐to‐clinical translation by providing comparable functional measures in animal models and humans. Animal models permit molecularly and genetically targeted mechanistic studies, as well as early testing of emerging therapies that are premature for human evaluation, such as bioelectronic interventions. Candidate therapies can then be evaluated using shared MRI endpoints across preclinical and clinical studies. This framework can help determine whether and how mechanisms and treatment effects observed in animal models translate to humans while identifying clinically important cross‐species differences.

## 7 Future Directions

To realize MRI's potential and utility for clinical practice, future efforts should further address current technical limitations and enhance mechanistic interpretability. A critical need is improved spatiotemporal resolution for reliable characterization of pyloric and duodenal functions. Although we envision dynamic MRI as a standalone, integrated diagnostic platform, validation against direct physiological measurements from inside the stomach remains important. Uniquely, simultaneous acquisition of dynamic MRI and high‐resolution APD manometry can relate wall kinematics and intraluminal pressure, providing deeper biomechanical insights for mechanistic interpretation. Utilizing 4‐D MRI data for CFD modeling will bridge the mechanistic gap and simulate the stomach in silico. Lastly, MRI is noninvasive and applicable to both animal models and human patients, supporting translation of findings from preclinical to clinical studies.

The field should unify protocols to ensure reproducibility, foster collaborations, and advance clinical adoption. This includes standardizing the composition of natural test meals, MRI acquisition parameters, and the analysis routines with clinician‐friendly software. Standardized, multi‐site MRI datasets will be crucial for training robust AI models for broader clinical applications.

Improving the management of gastroparesis and other disorders of gut‐brain interaction is not a winner‐takes‐all competition among diagnostic technologies. Existing and emerging methods, including BSGM and MRI, should be viewed as complementary. BSGM measures body‐surface electrical potentials and offers relatively accessible, prolonged recordings that may be easier to integrate into clinical practice. MRI is more resource‐intensive but provides high‐resolution, whole‐organ measurements of gastric morphology, wall kinematics, food transit, gastric accommodation, and emptying. Its defining strength is the direct assessment of regional motor function and interregional coordination. Potentially simultaneous acquisition of BGSM and MRI could relate body‐surface electrical potentials to underlying gastric motor events, laying the foundation to complement and enhance both modalities.

Routine clinical practice of dynamic gastric MRI will depend on the time, cost, scanner availability, as well as technical expertise, automated analysis, and reimbursement. Current protocols usually require imaging over 1–2 h after a test meal. This scanner occupancy time may limit the initial adoption, considering a typical hourly rate of $500 to $1000 for MRI scans in the United States. Although clinical MRI systems are widely available and sufficient for dynamic MRI, their access and affordability still vary across institutions and countries, and reimbursement pathways for comprehensive gastric MRI are not yet established. The pipeline for MRI acquisition and analysis should be standardized and automated to enable reliable and reproducible practice, which is important for broader adoption. An integrated MRI examination may offset some costs by consolidating assessments otherwise performed using multiple tests, but awaits formal cost‐effectiveness analyses. Towards the ultimate goal of establishing dynamic MRI as a clinic‐ready diagnostic platform, we include more detailed acquisition and analysis recommendations in the subsequent Appendix.

## Author Contributions

Zhongming Liu and Michael Camilleri conceptualized the paper. Xiaokai Wang and Zhongming Liu developed the MRI acquisition and analysis methods and drafted the manuscript. Michael Camilleri provided the clinical perspective and context. All authors critically revised the manuscript, approved the final version, and accept responsibility for the integrity of the work.

## Funding

This work was supported by National Institutes of Health, AT011665, DK131524, OD030538, OD023847.

## Conflicts of Interest

The authors declare no conflicts of interest.

## Appendix A

### MRI Acquisition and Analysis

Although a variety of pulse sequences can be applied to gastrointestinal MRI, recent studies converge on two pulse sequences for imaging gastric anatomy and motility [26, 27]. Anatomical scans use 2‐D balanced steady‐state free precession (bSSFP). With breath holding, 2‐D bSSFP can provide high‐contrast, high‐resolution details of gastric morphology and luminal contents for assessing gastric accommodation and emptying. However, its high specific absorption rate (SAR) limits the feasibility of continuous scans for motility assessment. Instead, motility (dynamic) scans use 3‐D gradient echo (GRE) with a small flip angle to capture T_1_‐weighted contrast that differentiates the ingested meal from surrounding tissues and organs. Given its lower SAR, 3‐D GRE can run repeatedly for 5–7 min with free breathing. Parallel imaging allows reconstruction with under‐sampled data from multiple coils, enabling < 3 s per frame to capture the full spectrum of gastric motility. Anatomical and motility scans can be interleaved and repeated every 10 min, typically for 1 or 2 h postprandially. Table A1 summarizes these pulse sequences, including their parameters, advantages, limitations, as well as practical recommendations.

**TABLE A1:** Summary of MRI pulse sequences to assess various aspects of gastric motor function, including parameter settings, advantages, limitations, and practical recommendations. Vendor‐specific acronyms for bSSFP and GRE are provided for Siemens, GE, and Philips.

Function	Pulse sequence	Parameters	Advantages	Limitations	Recommendations
Morphology	2‐D bSSFP Siemens: TrueFISP GE: FIESTA Philips: bFFE	TR: 3–5 ms TE: 1.2–2.5 ms FA: 50°–60° Bandwidth: 1200–1400 Hz/Px Slice orientation: axial Slice thickness: 3–6 mm Slice number: 20–40 Frequency: left–right Phase: anterior–posterior FOV: 360 × 270 mm^2^ Matrix: 256 × 192 Acceleration: 2 to 4 Fat saturation: No Total scan time: < 25 s	T_2_/T_1_ weighted contrast provides high contrast between the gastric wall and luminal content. Require no oral contrast, applicable both before and after meal. High SNR allows for high spatial resolution to resolve the gastric wall in the fasting condition and capture the gastric morphology and volume postprandially.	Require breath‐hold Susceptible to B0 inhomogeneity. High SAR precludes continuous scans for longer durations.	If a long breath hold is not feasible, scan two separate groups of slices during two breath holds. Scan immediately before and after the test meal to assess gastric accommodation. Repeat scans every 10 min to assess gastric emptying.
Accommodation					
Emptying					
Motility	3‐D GRE Siemens: VIBE GE: LAVA Philips: THRIVE	TR: 3–4 ms TE: 1–2 ms FA: 10°–15° Bandwidth: 350–500 Hz/Px Frequency: superior–inferior Phase: left–right Slice: anterior–posterior FOV: 360×270×144 Matrix: $192\times 144\times \left(32-44\right)$ Partial Fourier: 7/8 Acceleration: 3–5 Fat saturation: Yes Temporal resolution: < 3 s	T_1_‐weighted luminal contrast for rapid acquisition. Free‐breathing. < 3 s per frame to capture the full range of gastric motility. 3‐D volumetric acquisition of the complex and moving gastric morphology. Allow for re‐slicing. Lower SAR.	Require oral contrast. Moderate resolution to resolve the gastric wall. Respiratory motion artifacts. Moderate SNR. Need accelerated reconstruction with under‐sampled data.	Use a natural test meal, including pineapple. Continuous scan for 5 min to capture gastric motility. Interleave with anatomical scans every 7–10 min. Prioritize temporal resolution over spatial resolution. Record respiration, heart rate, and EGG, and raw data for retrospective reconstruction and analysis.
Pylorus					

Dynamic MRI creates large videos of the moving stomach. However, the need for manual or semi‐automated analysis is still a critical bottleneck. Recent studies have made progress towards a fully automated analysis pipeline, including respiratory motion correction, image segmentation, modeling surface kinematics, and mapping motility features [23, 26, 27, 28, 29, 33, 186].

Respiratory motion may corrupt data in k‐space and confound gastric motion in image space. Although fast acquisition reduces k‐space corruption, intermittent ghosting artifacts still occur. Recording the respiratory signal enables correction of the k‐space data based on the respiratory phase of each time frame [29]. In the image space, respiration‐induced diaphragmatic motion causes bulk displacement to the stomach, whereas intrinsic gastric motion occurs at much finer spatial scales. To decouple them, one approach is to first estimate the bulk displacement using large‐scale nonlinear registration and then remove the estimated displacement to preserve local gastric motion [27].

The next step is to segment the stomach from the surrounding organs. A traditional method segments the intragastric lumen by thresholding the image intensity [23, 26, 27]; however, the results are susceptible to inhomogeneous distributions inside the stomach and require manual correction. A new method uses a pre‐trained AI model, SAM‐2 [187], to automatically segment a dynamic series of images given a prompt that supplies a rough segmentation of only the first image in the series; this method yields significantly improved efficiency and throughput [186].

Once the gastric volume is segmented, a 3‐D surface template of the stomach is algorithmically deformed to enclose the segmented volume and capture the time‐varying gastric morphology. Gastric wall motions are modeled by diffeomorphic transformations through a neural ordinary differential equation (ODE) [28]. In this model, the surface mesh serves as a scaffold for the gastric musculature. Its diffeomorphic transformations preserve the topology while capturing the biomechanics of gastric muscles. By quantifying motion at every surface location as well as local areal change, this analysis effectively extracts and maps a comprehensive set of quantitative motor metrics. Periodic changes characterize phasic contractile waves, including their initiation, propagation, amplitude, frequency, and velocity. Sustained changes reflect tonic elongation or shortening of muscle fibers for gastric accommodation or emptying, respectively [26]. Uniquely, the use of a surface template allows direct comparison of motility within and between individuals [26].

## References

[1] R. K. Goyal , Y. Guo , and H. Mashimo , “Advances in the Physiology of Gastric Emptying,” Neurogastroenterology and Motility 31, no. 4 (2019): e13546, 10.1111/nmo.13546. PMC6850045 30740834

[2] M. Camilleri and K. M. Sanders , “Gastroparesis,” Gastroenterology 162, no. 1 (2022): 68–87, 10.1053/j.gastro.2021.10.028. PMC8678360 34717924

[3] P. J. Pasricha and H. P. Parkman , “Gastroparesis: definitions and diagnosis,” Gastroenterology Clinics of North America 44, no. 1 (2015): 1–7, 10.1016/j.gtc.2014.11.001. 25667018

[4] M. Camilleri , B. Kuo , L. Nguyen , et al., “ACG Clinical Guideline: Gastroparesis,” American Journal of Gastroenterology 117, no. 8 (2022): 1197–1220, 10.14309/ajg.0000000000001874. PMC9373497 35926490

[5] M. Camilleri , V. Chedid , A. C. Ford , et al., “Gastroparesis,” Nature Reviews Disease Primers 4, no. 1 (2018): 41, 10.1038/s41572-018-0038-z. 30385743

[6] W. L. Hasler , “Gastroparesis: Pathogenesis, Diagnosis and Management,” Nature Reviews 8, no. 8 (2011): 438–453, 10.1038/nrgastro.2011.116. 21769117

[7] Y. Ye , Y. Yin , S. Y. Huh , C. Almansa , D. Bennett , and M. Camilleri , “Epidemiology, Etiology, and Treatment of Gastroparesis: Real‐World Evidence From a Large US National Claims Database,” Gastroenterology 162, no. 1 (2022): 109–121.e5, 10.1053/j.gastro.2021.09.064. 34624355

[8] S. Dilmaghani , T. Zheng , and M. Camilleri , “Epidemiology and Healthcare Utilization in Patients With Gastroparesis: A Systematic Review,” Clinical Gastroenterology and Hepatology 21, no. 9 (2023): 2239–2251.e2, 10.1016/j.cgh.2022.07.011. PMC9852358 35870768

[9] V. Wadhwa , D. Mehta , Y. Jobanputra , R. Lopez , P. N. Thota , and M. R. Sanaka , “Healthcare Utilization and Costs Associated With Gastroparesis,” World Journal of Gastroenterology 23, no. 24 (2017): 4428–4436, 10.3748/wjg.v23.i24.4428. PMC5487507 28706426

[10] M. Camilleri , H. P. Parkman , M. A. Shafi , T. L. Abell , and L. Gerson , “Clinical Guideline: Management of Gastroparesis,” Official Journal of the American College of Gastroenterology | ACG 108, no. 1 (2013): 18, 10.1038/ajg.2012.373. PMC3722580 23147521

[11] H. P. Parkman , W. L. Hasler , R. S. Fisher , and American Gastroenterological Association , “American Gastroenterological Association Technical Review on the Diagnosis and Treatment of Gastroparesis,” Gastroenterology 127, no. 5 (2004): 1592–1622, 10.1053/j.gastro.2004.09.055. 15521026

[12] S. Y. Park , A. Acosta , M. Camilleri , et al., “Gastric Motor Dysfunction in Patients With Functional Gastroduodenal Symptoms,” American Journal of Gastroenterology 112, no. 11 (2017): 1689–1699, 10.1038/ajg.2017.264. 28895582

[13] M. Camilleri , J. R. Malagelada , M. L. Brown , G. Becker , and A. R. Zinsmeister , “Relation Between Antral Motility and Gastric Emptying of Solids and Liquids in Humans,” American Journal of Physiology 249, no. 5 Pt 1 (1985): G580–G585, 10.1152/ajpgi.1985.249.5.G580. 4061646

[14] T. Zheng , J. BouSaba , W. Sannaa , D. J. Eckert , D. D. Burton , and M. Camilleri , “Comprehensive Characterization of Antral and Pyloric Contractions by High Resolution Manometry: Applied Physiology in Suspected Gastroparesis,” American Journal of Physiology 323, no. 3 (2022): G255–G264, 10.1152/ajpgi.00119.2022. PMC9448275 35819155

[15] F. Mearin , M. Camilleri , and J. R. Malagelada , “Pyloric Dysfunction in Diabetics With Recurrent Nausea and Vomiting,” Gastroenterology 90, no. 6 (1986): 1919–1925, 10.1016/0016-5085(86)90262-3. 3699409

[16] Z. Malik , A. Sankineni , and H. P. Parkman , “Assessing Pyloric Sphincter Pathophysiology Using EndoFLIP in Patients With Gastroparesis,” Neurogastroenterology and Motility 27, no. 4 (2015): 524–531, 10.1111/nmo.12522. 25712043

[17] F. Wuestenberghs and G. Gourcerol , “Pyloric Distensibility in Health and Disease,” American Journal of Physiology. Gastrointestinal and Liver Physiology 321, no. 2 (2021): G133–G138, 10.1152/ajpgi.00460.2020. 34160292

[18] M. Camilleri , M. L. Brown , and J. R. Malagelada , “Relationship Between Impaired Gastric Emptying and Abnormal Gastrointestinal Motility,” Gastroenterology 91, no. 1 (1986): 94–99, 10.1016/0016-5085(86)90444-0. 3710086

[19] E. L. Calvert , P. J. Whorwell , and L. A. Houghton , “Inter‐Digestive and Post‐Prandial Antro‐Pyloro‐Duodenal Motor Activity in Humans: Effect of 5‐Hydroxytryptamine 1 Receptor Agonism,” Alimentary Pharmacology & Therapeutics 19, no. 7 (2004): 805–815, 10.1111/j.1365-2036.2004.01901.x. 15043522

[20] P. Orthey , S. Dadparvar , B. Kamat , H. P. Parkman , and A. H. Maurer , “Using Gastric Emptying Scintigraphy to Evaluate Antral Contractions and Duodenal Bolus Propagation,” American Journal of Physiology. Gastrointestinal and Liver Physiology 318, no. 1 (2020): G203–G209, 10.1152/ajpgi.00274.2019. 31682161

[21] T. L. Powley , “Brain‐Gut Communication: Vagovagal Reflexes Interconnect the Two Brains,” American Journal of Physiology. Gastrointestinal and Liver Physiology 321, no. 5 (2021): G576–G587, 10.1152/ajpgi.00214.2021. PMC8616589 34643086

[22] J. B. Furness , M. Di Natale , B. Hunne , et al., “The Identification of Neuronal Control Pathways Supplying Effector Tissues in the Stomach,” Cell and Tissue Research 382, no. 3 (2020): 433–445, 10.1007/s00441-020-03294-7. PMC7727782 33156383

[23] K. H. Lu , J. Cao , S. T. Oleson , T. L. Powley , and Z. Liu , “Contrast‐Enhanced Magnetic Resonance Imaging of Gastric Emptying and Motility in Rats,” IEEE Transactions on Biomedical Engineering 64, no. 11 (2017): 2546–2554, 10.1109/TBME.2017.2737559. PMC7439606 28796602

[24] F. Maccioni , L. Busato , A. Valenti , S. Cardaccio , A. Longhi , and C. Catalano , “Magnetic Resonance Imaging of the Gastrointestinal Tract: Current Role, Recent Advancements and Future Prospectives,” Diagnostics 13, no. 14 (2023): 2410, 10.3390/diagnostics13142410. PMC10378103 37510154

[25] R. Sclocco , C. Nguyen , R. Staley , et al., “Non‐Uniform Gastric Wall Kinematics Revealed by 4D Cine Magnetic Resonance Imaging in Humans,” Neurogastroenterology and Motility 33, no. 8 (2021): e14146, 10.1111/nmo.14146. PMC10315015 33797166

[26] X. Wang , F. Alkaabi , M. Choi , et al., “Surface Mapping of Gastric Motor Functions Using MRI: A Comparative Study Between Humans and Rats,” American Journal of Physiology. Gastrointestinal and Liver Physiology 327, no. 3 (2024): G345–G359, 10.1152/ajpgi.00045.2024. PMC11427095 38915290

[27] K. H. Lu , Z. Liu , D. Jaffey , et al., “Automatic Assessment of Human Gastric Motility and Emptying From Dynamic 3D Magnetic Resonance Imaging,” Neurogastroenterology and Motility 34, no. 1 (2022): e14239, 10.1111/nmo.14239. 34431171

[28] X. Wang , J. Cao , K. Han , et al., “Diffeomorphic Surface Modeling for MRI‐Based Characterization of Gastric Anatomy and Motility,” IEEE Transactions on Biomedical Engineering 70, no. 7 (2023): 2046–2057, 10.1109/TBME.2023.3234509. PMC10443119 37018592

[29] X. Wang , F. Alkaabi , A. Cornett , et al., “Magnetic Resonance Imaging of Gastric Motility in Conscious Rats,” Neurogastroenterology and Motility 38, no. 1 (2026): e14982, 10.1111/nmo.14982. PMC12206934 39737873

[30] M. Chavero‐Pieres , M. F. Viola , I. Appeltans , et al., “Magnetic Resonance Imaging as a Non‐Invasive Tool to Assess Gastric Emptying in Mice,” Neurogastroenterology and Motility 35, no. 2 (2023): e14490, 10.1111/nmo.14490. PMC10078537 36371706

[31] K. H. Lu , J. Cao , R. Phillips , T. L. Powley , and Z. Liu , “Acute Effects of Vagus Nerve Stimulation Parameters on Gastric Motility Assessed With Magnetic Resonance Imaging,” Neurogastroenterology and Motility 32, no. 7 (2020): e13853, 10.1111/nmo.13853. PMC7872206 32297404

[32] K. H. Lu , J. Cao , S. Oleson , et al., “Vagus Nerve Stimulation Promotes Gastric Emptying by Increasing Pyloric Opening Measured With Magnetic Resonance Imaging,” Neurogastroenterology and Motility 30, no. 10 (2018): e13380, 10.1111/nmo.13380. PMC6160317 29797377

[33] X. Wang , U. M. Scheven , and Z. Liu , “Dynamic Magnetic Resonance Imaging of Whole‐Stomach Motility in Rats,” NMR in Biomedicine 38, no. 10 (2025): e70138, 10.1002/nbm.70138. PMC12421216 40926545

[34] L. Marciani , P. A. Gowland , R. C. Spiller , et al., “Effect of Meal Viscosity and Nutrients on Satiety, Intragastric Dilution, and Emptying Assessed by MRI,” American Journal of Physiology. Gastrointestinal and Liver Physiology 280, no. 6 (2001): G1227–G1233, 10.1152/ajpgi.2001.280.6.G1227. 11352816

[35] L. Marciani , P. Young , J. Wright , et al., “Antral Motility Measurements by Magnetic Resonance Imaging,” Neurogastroenterology and Motility 13, no. 5 (2001): 511–518, 10.1046/j.1365-2982.2001.00285.x. 11696113

[36] M. R. Di Natale , O. N. Athavale , X. Wang , et al., “Functional and Anatomical Gastric Regions and Their Relations to Motility Control,” Neurogastroenterology and Motility 35, no. 9 (2023): e14560, 10.1111/nmo.14560. 36912719

[37] M. Camilleri , “Gastrointestinal Hormones and Regulation of Gastric Emptying,” Current Opinion in Endocrinology, Diabetes, and Obesity 26, no. 1 (2019): 3–10, 10.1097/MED.0000000000000448. PMC6615897 30418188

[38] S. Rao , C. Lu , and K. Schulze‐Delrieu , “Duodenum as a Immediate Brake to Gastric Outflow: A Videofluoroscopic and Manometric Assessment,” Gastroenterology 110, no. 3 (1996): 740–747, 10.1053/gast.1996.v110.pm8608883. 8608883

[39] M. R. Di Natale , X. Wang , B. Hunne , et al., “Regional Specialisations of Innervation and Musculature of the Rat Stomach,” Journal of Anatomy 249 (2026): 634–651, 10.1111/joa.70105. PMC13398461 41470075

[40] J. B. Furness , U. Marklund , B. J. Czapla , J. C. Bornstein , and M. N. Han , “Functional Characterization and Classification of Enteric Neurons, and Regional Differences in Neural Control of Digestive Functions,” Autonomic Neuroscience 265 (2026): 103412, 10.1016/j.autneu.2026.103412. 41855601

[41] J. B. Furness , “The Enteric Nervous System and Neurogastroenterology,” Nature Reviews. Gastroenterology & Hepatology 9, no. 5 (2012): 286–294, 10.1038/nrgastro.2012.32. 22392290

[42] M. R. Di Natale , L. Patten , J. C. Molero , et al., “Organisation of the Musculature of the Rat Stomach,” Journal of Anatomy 240, no. 4 (2022): 711–723, 10.1111/joa.13587. PMC8930815 34747011

[43] M. R. Di Natale , B. Hunne , M. J. Stebbing , X. Wang , Z. Liu , and J. B. Furness , “Characterization of Neuromuscular Transmission and Projections of Muscle Motor Neurons in the Rat Stomach,” American Journal of Physiology. Gastrointestinal and Liver Physiology 326, no. 1 (2024): G78–G93, 10.1152/ajpgi.00194.2023. PMC11208016 37987773

[44] K. M. Sanders , S. M. Ward , and S. D. Koh , “Interstitial Cells: Regulators of Smooth Muscle Function,” Physiological Reviews 94, no. 3 (2014): 859–907, 10.1152/physrev.00037.2013. PMC4152167 24987007

[45] S. M. Ward , E. A. Beckett , X. Wang , F. Baker , M. Khoyi , and K. M. Sanders , “Interstitial Cells of Cajal Mediate Cholinergic Neurotransmission From Enteric Motor Neurons,” Journal of Neuroscience 20, no. 4 (2000): 1393–1403, 10.1523/JNEUROSCI.20-04-01393.2000. PMC6772355 10662830

[46] P. J. Blair , Y. Bayguinov , K. M. Sanders , and S. M. Ward , “Relationship Between Enteric Neurons and Interstitial Cells in the Primate Gastrointestinal Tract,” Neurogastroenterology and Motility 24, no. 9 (2012): e437–e449, 10.1111/j.1365-2982.2012.01975.x. PMC4854185 22805588

[47] A. J. Burns , A. E. Lomax , S. Torihashi , K. M. Sanders , and S. M. Ward , “Interstitial Cells of Cajal Mediate Inhibitory Neurotransmission in the Stomach,” Proceedings of the National Academy of Sciences of the United States of America 93, no. 21 (1996): 12008–12013, 10.1073/pnas.93.21.12008. PMC38174 8876253

[48] S. J. Hwang , J. G. Kwon , E. A. H. Beckett , et al., “Functional Roles of Interstitial Cells of Cajal in the GI Tract of Rats,” American Journal of Physiology – Gastrointestinal and Liver Physiology 328, no. 6 (2025): G677–G695, 10.1152/ajpgi.00036.2025. PMC12747247 40235202

[49] K. Sanders , “A Case for Interstitial Cells of Cajal as Pacemakers and Mediators of Neurotransmission in the Gastrointestinal Tract,” Gastroenterology 111, no. 2 (1996): 492–515, 10.1053/gast.1996.v111.pm8690216. 8690216

[50] K. M. Sanders , S. D. Koh , and S. M. Ward , “Interstitial Cells of CAJAL as Pacemakers in the Gastrointestinal Tract,” Annual Review of Physiology 68 (2006): 307–343, 10.1146/annurev.physiol.68.040504.094718. 16460275

[51] L. Thomsen , T. L. Robinson , J. C. Lee , et al., “Interstitial Cells of Cajal Generate a Rhythmic Pacemaker Current,” Nature Medicine 4, no. 7 (1998): 848–851, 10.1038/nm0798-848. 9662380

[52] S. Y. Zhou , Y. X. Lu , H. Yao , and C. Owyang , “Spatial Organization of Neurons in the Dorsal Motor Nucleus of the Vagus Synapsing With Intragastric Cholinergic and Nitric Oxide/VIP Neurons in the Rat,” American Journal of Physiology. Gastrointestinal and Liver Physiology 294, no. 5 (2008): G1201–G1209, 10.1152/ajpgi.00309.2006. PMC3221413 18460697

[53] D. M. Jaffey , J. L. McAdams , E. A. Baronowsky , D. Black , and T. L. Powley , “Vagal Preganglionic Axons Arborize in the Myenteric Plexus Into Two Types: Nitrergic and Non‐Nitrergic Postganglionic Motor Pools?,” American Journal of Physiology. Regulatory, Integrative and Comparative Physiology 324, no. 3 (2023): R305–R316, 10.1152/ajpregu.00260.2022. PMC9942884 36622086

[54] R. A. Travagli and L. Anselmi , “Vagal Neurocircuitry and Its Influence on Gastric Motility,” Nature Reviews. Gastroenterology & Hepatology 13, no. 7 (2016): 389–401, 10.1038/nrgastro.2016.76. PMC5605144 27251213

[55] T. L. Powley , D. M. Jaffey , J. McAdams , et al., “Vagal Innervation of the Stomach Reassessed: Brain−Gut Connectome Uses Smart Terminals,” Annals of the New York Academy of Sciences 1454, no. 1 (2019): 14–30, 10.1111/nyas.14138. PMC6810743 31268562

[56] Y. B. Wang , G. de Lartigue , and A. J. Page , “Dissecting the Role of Subtypes of Gastrointestinal Vagal Afferents,” Frontiers in Physiology 11 (2020): 643, 10.3389/fphys.2020.00643. PMC7300233 32595525

[57] V. P. Zagorodnyuk , B. N. Chen , and S. J. H. Brookes , “Intraganglionic Laminar Endings Are Mechano‐Transduction Sites of Vagal Tension Receptors in the Guinea‐Pig Stomach,” Journal of Physiology 534, no. Pt 1 (2001): 255–268, 10.1111/j.1469-7793.2001.00255.x. PMC2278677 11433006

[58] T. L. Powley and R. J. Phillips , “Vagal Intramuscular Array Afferents Form Complexes With Interstitial Cells of Cajal in Gastrointestinal Smooth Muscle: Analogues of Muscle Spindle Organs?,” Neuroscience 186 (2011): 188–200, 10.1016/j.neuroscience.2011.04.036. PMC3110524 21530617

[59] R. J. Phillips and T. L. Powley , “Tension and Stretch Receptors in Gastrointestinal Smooth Muscle: Re‐Evaluating Vagal Mechanoreceptor Electrophysiology,” Brain Research Reviews 34, no. 1 (2000): 1–26, 10.1016/S0165-0173(00)00036-9. 11086184

[60] J. Cao , X. Wang , T. L. Powley , and Z. Liu , “Gastric Neurons in the Nucleus Tractus Solitarius Are Selective to the Orientation of Gastric Electrical Stimulation,” Journal of Neural Engineering 18, no. 5 (2021): 056066, 10.1088/1741-2552/ac2ec6. PMC8625070 34634781

[61] C. Ran , J. C. Boettcher , J. A. Kaye , C. E. Gallori , and S. D. Liberles , “A Brainstem Map for Visceral Sensations,” Nature 609, no. 7926 (2022): 320–326, 10.1038/s41586-022-05139-5. PMC9452305 36045291

[62] S. Oleson , J. Cao , X. Wang , and Z. Liu , “In Vivo Tracing of the Ascending Vagal Projections to the Brain With Manganese Enhanced Magnetic Resonance Imaging,” Frontiers in Neuroscience 17 (2023): 1254097, 10.3389/fnins.2023.1254097. PMC10540305 37781260

[63] J. Cao , X. Wang , J. Chen , N. Zhang , and Z. Liu , “The Vagus Nerve Mediates the Stomach‐Brain Coherence in Rats,” NeuroImage 263 (2022): 119628, 10.1016/j.neuroimage.2022.119628. PMC10008817 36113737

[64] J. Cao , K. H. Lu , S. T. Oleson , et al., “Gastric Stimulation Drives Fast BOLD Responses of Neural Origin,” NeuroImage 197 (2019): 200–211, 10.1016/j.neuroimage.2019.04.064. PMC6594550 31029867

[65] K. N. Browning and R. A. Travagli , “Central Nervous System Control of Gastrointestinal Motility and Secretion and Modulation of Gastrointestinal Functions,” Comprehensive Physiology 4, no. 4 (2014): 1339–1368, 10.1002/cphy.c130055. PMC4858318 25428846

[66] W. Han , L. A. Tellez , M. H. Perkins , et al., “A Neural Circuit for Gut‐Induced Reward,” Cell 175, no. 3 (2018): 665–678.e23, 10.1016/j.cell.2018.08.049. PMC6195474 30245012

[67] M. M. Kaelberer , K. L. Buchanan , M. E. Klein , et al., “A gut‐brain neural circuit for nutrient sensory transduction,” Science 361, no. 6408 (2018): eaat5236, 10.1126/science.aat5236. PMC6417812 30237325

[68] T. Zhang , M. H. Perkins , H. Chang , W. Han , and I. E. de Araujo , “An inter‐organ neural circuit for appetite suppression,” Cell 185, no. 14 (2022): 2478–2494.e28, 10.1016/j.cell.2022.05.007. PMC9433108 35662413

[69] D. Ramkumar and K. S. Schulze , “The Pylorus,” Neurogastroenterology and Motility 17, no. s1 (2005): 22–30, 10.1111/j.1365-2982.2005.00664.x. 15836452

[70] X. J. Wang , D. D. Burton , M. Breen‐Lyles , and M. Camilleri , “Gastric Accommodation Influences Proximal Gastric and Total Gastric Emptying in Concurrent Measurements Conducted in Healthy Volunteers,” American Journal of Physiology. Gastrointestinal and Liver Physiology 320, no. 5 (2021): G759–G767, 10.1152/ajpgi.00008.2021. PMC8202200 33719546

[71] K. Indireshkumar , J. G. Brasseur , H. Faas , et al., “Relative Contributions of “Pressure Pump” and “Peristaltic Pump” to Gastric Emptying,” American Journal of Physiology. Gastrointestinal and Liver Physiology 278, no. 4 (2000): G604–G616, 10.1152/ajpgi.2000.278.4.G604. 10762615

[72] M. A. Kwiatek , D. Menne , A. Steingoetter , et al., “Effect of Meal Volume and Calorie Load on Postprandial Gastric Function and Emptying: Studies Under Physiological Conditions by Combined Fiber‐Optic Pressure Measurement and MRI,” American Journal of Physiology. Gastrointestinal and Liver Physiology 297, no. 5 (2009): G894–G901, 10.1152/ajpgi.00117.2009. 19779010

[73] K. M. Sanders , S. D. Koh , S. Ro , and S. M. Ward , “Regulation of Gastrointestinal Motility—Insights From Smooth Muscle Biology,” Nature Reviews. Gastroenterology & Hepatology 9, no. 11 (2012): 633–645, 10.1038/nrgastro.2012.168. PMC4793911 22965426

[74] G. O'Grady , P. Du , L. K. Cheng , et al., “Origin and Propagation of Human Gastric Slow‐Wave Activity Defined by High‐Resolution Mapping,” American Journal of Physiology. Gastrointestinal and Liver Physiology 299, no. 3 (2010): G585–G592, 10.1152/ajpgi.00125.2010. PMC2950696 20595620

[75] S. A. Baker , S. J. Hwang , P. J. Blair , et al., “Ca^2+^ Transients in ICC‐MY Define the Basis for the Dominance of the Corpus in Gastric Pacemaking,” Cell Calcium 99 (2021): 102472, 10.1016/j.ceca.2021.102472. PMC8592010 34537580

[76] M. Bashashati and R. W. McCallum , “Is Interstitial Cells of Cajal–Opathy Present in Gastroparesis?,” Journal of Neurogastroenterology and Motility 21, no. 4 (2015): 486–493, 10.5056/jnm15075. PMC4622130 26424037

[77] E. Maalouf , X. y. Chai , J. A. Patraw , et al., “Changes in Interstitial Cells of Cajal Calcium Activity in a Diabetic Mouse Model of Delayed Gastric Emptying,” Journal of Physiology 604, no. 14 (2026): 5800–5816, 10.1113/JP290718. PMC13370688 42218355

[78] G. Farrugia , “Interstitial Cells of Cajal in Health and Disease,” Neurogastroenterology and Motility 20, no. Suppl 1 (2008): 54–63, 10.1111/j.1365-2982.2008.01109.x. 18402642

[79] T. R. Angeli , L. K. Cheng , P. Du , et al., “Loss of Interstitial Cells of Cajal and Patterns of Gastric Dysrhythmia in Patients With Chronic Unexplained Nausea and Vomiting,” Gastroenterology 149, no. 1 (2015): 56–66, 10.1053/j.gastro.2015.04.003. PMC4617790 25863217

[80] G. O'Grady , T. R. Angeli , P. Du , et al., “Abnormal Initiation and Conduction of Slow‐Wave Activity in Gastroparesis, Defined by High‐Resolution Electrical Mapping,” Gastroenterology 143, no. 3 (2012): 589–598, 10.1053/j.gastro.2012.05.036. PMC3429650 22643349

[81] K. L. Koch , “Gastric Dysrhythmias: A Potential Objective Measure of Nausea,” Experimental Brain Research 232, no. 8 (2014): 2553–2561, 10.1007/s00221-014-4007-9. 24916149

[82] M. J. Vassallo , M. Camilleri , C. M. Prather , R. B. Hanson , and G. M. Thomforde , “Measurement of Axial Forces During Emptying From the Human Stomach,” American Journal of Physiology 263, no. 2 Pt 1 (1992): G230–G239, 10.1152/ajpgi.1992.263.2.G230. 1514635

[83] M. Camilleri and C. M. Prather , “Axial Forces During Gastric Emptying in Health and Models of Disease,” Digestive Diseases and Sciences 39, no. 12 Suppl (1994): 14S–17S, 10.1007/BF02300361. 7995207

[84] S. Kuhar , J. H. Seo , and R. Mittal , “In Silico Study of the Dynamics of Solid Food Particles in the Stomach During Gastric Digestion,” Journal of the Royal Society Interface 22, no. 229 (2025): 20250291, 10.1098/rsif.2025.0291. PMC12364583 40829641

[85] S. R. Vavricka and T. Greuter , “Gastroparesis and Dumping Syndrome: Current Concepts and Management,” Journal of Clinical Medicine 8, no. 8 (2019): 1127, 10.3390/jcm8081127. PMC6723467 31362413

[86] H. Soliman and G. Gourcerol , “Targeting the Pylorus in Gastroparesis: From Physiology to Endoscopic Pyloromyotomy,” Neurogastroenterology and Motility 35, no. 2 (2023): e14529, 10.1111/nmo.14529. PMC10077918 36594414

[87] M. Castedal , E. Björnsson , and H. Abrahamsson , “Postprandial Peristalsis in the Human Duodenum,” Neurogastroenterology and Motility 10, no. 3 (1998): 227–233, 10.1046/j.1365-2982.1998.00098.x. 9659666

[88] S. Kuhar , J. H. Seo , P. J. Pasricha , M. Camilleri , and R. Mittal , “Duodenogastric Reflux in Health and Disease: Insights From a Computational Fluid Dynamics Model of the Stomach,” American Journal of Physiology. Gastrointestinal and Liver Physiology 328, no. 4 (2025): G411–G425, 10.1152/ajpgi.00241.2024. 39873302

[89] R. A. Liddle , E. T. Morita , C. K. Conrad , and J. A. Williams , “Regulation of Gastric Emptying in Humans by Cholecystokinin,” Journal of Clinical Investigation 77, no. 3 (1986): 992–996, 10.1172/JCI112401. PMC423501 3949984

[90] M. A. Nauck , U. Niedereichholz , R. Ettler , et al., “Glucagon‐Like Peptide 1 Inhibition of Gastric Emptying Outweighs Its Insulinotropic Effects in Healthy Humans,” American Journal of Physiology 273, no. 5 (1997): E981–E988, 10.1152/ajpendo.1997.273.5.E981. 9374685

[91] A. P. Savage , T. E. Adrian , G. Carolan , V. K. Chatterjee , and S. R. Bloom , “Effects of Peptide YY (PYY) on Mouth to Caecum Intestinal Transit Time and on the Rate of Gastric Emptying in Healthy Volunteers,” Gut 28, no. 2 (1987): 166–170, 10.1136/gut.28.2.166. PMC1432980 3557189

[92] M. Camilleri , “Integrated Upper Gastrointestinal Response to Food Intake,” Gastroenterology 131, no. 2 (2006): 640–658, 10.1053/j.gastro.2006.03.023. 16890616

[93] T. L. Abell , M. Camilleri , K. Donohoe , et al., “Consensus Recommendations for Gastric Emptying Scintigraphy: A Joint Report of the American Neurogastroenterology and Motility Society and the Society of Nuclear Medicine,” Journal of Nuclear Medicine Technology 36, no. 1 (2008): 44–54, 10.2967/jnmt.107.048116. 18287197

[94] M. Camilleri , J. Iturrino , A. E. Bharucha , et al., “Performance Characteristics of Scintigraphic Measurement of Gastric Emptying of Solids in Healthy Participants,” Neurogastroenterology and Motility 24, no. 12 (2012): 1076, 10.1111/j.1365-2982.2012.01972.x. PMC3465511 22747676

[95] S. T. Odunsi , M. Camilleri , L. A. Szarka , and A. R. Zinsmeister , “Optimizing Analysis of Stable Isotope Breath Tests to Estimate Gastric Emptying of Solids,” Neurogastroenterology and Motility 21, no. 7 (2009): 706, 10.1111/j.1365-2982.2009.01283.x. PMC2704250 19309440

[96] L. A. Szarka , M. Camilleri , A. Vella , et al., “A Stable Isotope Breath Test With a Standard Meal for Abnormal Gastric Emptying of Solids in the Clinic and in Research,” Clinical Gastroenterology and Hepatology 6, no. 6 (2008): 635–643.e1, 10.1016/j.cgh.2008.01.009. PMC3739971 18406670

[97] J. L. C. Urbain , M. C. Vekemans , H. Parkman , et al., “Dynamic Antral Scintigraphy to Characterize Gastric Antral Motility in Functional Dyspepsia,” Journal of Nuclear Medicine 36, no. 9 (1995): 1579–1586. 7658213

[98] A. H. Maurer , P. Silver , D. Yu , et al., “Fourier Phase Analysis of Dynamic Antral Contraction Scintigraphy: New Software, Reference Values, and Comparisons to Conventional Gastric Emptying,” Journal of Nuclear Medicine Technology 52, no. 1 (2024): 32–39, 10.2967/jnmt.122.265037. 36750378

[99] J. Tack , H. Piessevaux , B. Coulie , P. Caenepeel , and J. Janssens , “Role of Impaired Gastric Accommodation to a Meal in Functional Dyspepsia,” Gastroenterology 115, no. 6 (1998): 1346–1352, 10.1016/s0016-5085(98)70012-5. 9834261

[100] F. Azpiroz and J. R. Malagelada , “Gastric Tone Measured by an Electronic Barostat in Health and Postsurgical Gastroparesis,” Gastroenterology 92, no. 4 (1987): 934–943, 10.1016/0016-5085(87)90967-x. 3556999

[101] M. W. Mundt , T. Hausken , and M. Samsom , “Effect of Intragastric Barostat Bag on Proximal and Distal Gastric Accommodation in Response to Liquid Meal,” American Journal of Physiology. Gastrointestinal and Liver Physiology 283, no. 3 (2002): G681–G686, 10.1152/ajpgi.00499.2001. 12181183

[102] I. M. de Zwart , J. J. L. Haans , P. Verbeek , P. H. C. Eilers , A. de Roos , and A. A. M. Masclee , “Gastric Accommodation and Motility Are Influenced by the Barostat Device: Assessment With Magnetic Resonance Imaging,” American Journal of Physiology. Gastrointestinal and Liver Physiology 292, no. 1 (2007): G208–G214, 10.1152/ajpgi.00151.2006. 16891299

[103] E. P. Bouras , S. Delgado‐Aros , M. Camilleri , et al., “SPECT Imaging of the Stomach: Comparison With Barostat, and Effects of Sex, Age, Body Mass Index, and Fundoplication. Single Photon Emission Computed Tomography,” Gut 51, no. 6 (2002): 781–786, 10.1136/gut.51.6.781. PMC1773479 12427776

[104] S. D. Kuiken , M. Samsom , M. Camilleri , et al., “Development of a Test to Measure Gastric Accommodation in Humans,” American Journal of Physiology 277, no. 6 (1999): G1217–G1221, 10.1152/ajpgi.1999.277.6.G1217. 10600819

[105] O. H. Gilja , T. Hausken , I. Wilhelmsen , and A. Berstad , “Impaired Accommodation of Proximal Stomach to a Meal in Functional Dyspepsia,” Digestive Diseases and Sciences 41, no. 4 (1996): 689–696, 10.1007/BF02213124. 8674389

[106] O. H. Gilja , J. Lunding , T. Hausken , and H. Gregersen , “Gastric Accommodation Assessed by Ultrasonography,” World Journal of Gastroenterology 12, no. 18 (2006): 2825–2829, 10.3748/wjg.v12.i18.2825. PMC4087797 16718805

[107] M. L. Manini , D. D. Burton , D. D. Meixner , et al., “Feasibility and Application of 3‐Dimensional Ultrasound for Measurement of Gastric Volumes in Healthy Adults and Adolescents,” Journal of Pediatric Gastroenterology and Nutrition 48, no. 3 (2009): 287–293, 10.1097/mpg.0b013e318189694f. PMC2654599 19274782

[108] M. Camilleri , A. E. Bharucha , C. Di Lorenzo , et al., “American Neurogastroenterology and Motility Society Consensus Statement on Intraluminal Measurement of Gastrointestinal and Colonic Motility in Clinical Practice,” Neurogastroenterology and Motility 20, no. 12 (2008): 1269–1282, 10.1111/j.1365-2982.2008.01230.x. 19019032

[109] J. Desipio , F. K. Friedenberg , A. Korimilli , J. E. Richter , H. P. Parkman , and R. S. Fisher , “High‐Resolution Solid‐State Manometry of the Antropyloroduodenal Region,” Neurogastroenterology and Motility 19, no. 3 (2007): 188–195, 10.1111/j.1365-2982.2006.00866.x. 17300288

[110] A. D. Nelson , M. Camilleri , A. Acosta , et al., “Effects of Ghrelin Receptor Agonist, Relamorelin, on Gastric Motor Functions and Satiation in Healthy Volunteers,” Neurogastroenterology and Motility 28, no. 11 (2016): 1705–1713, 10.1111/nmo.12870. PMC5083171 27283792

[111] H. Soliman , F. Wuestenberghs , C. Desprez , A. M. Leroi , C. Melchior , and G. Gourcerol , “Physiological Characterization of Gastric Emptying Using High‐Resolution Antropyloroduodenal Manometry,” American Journal of Physiology. Gastrointestinal and Liver Physiology 326, no. 1 (2024): G16–G24, 10.1152/ajpgi.00101.2023. 37874655

[112] G. Parthasarathy , K. Ravi , M. Camilleri , et al., “Effect of Neostigmine on Gastroduodenal Motility in Patients With Suspected Gastrointestinal Motility Disorders,” Neurogastroenterology and Motility 27, no. 12 (2015): 1736–1746, 10.1111/nmo.12669. PMC4659742 26387781

[113] N. S. Coleman , L. Marciani , E. Blackshaw , et al., “Effect of a Novel 5‐HT3 Receptor Agonist MKC‐733 on Upper Gastrointestinal Motility in Humans,” Alimentary Pharmacology & Therapeutics 18, no. 10 (2003): 1039–1048, 10.1046/j.1365-2036.2003.01797.x. 14616171

[114] R. Rosen , J. M. Garza , N. Tipnis , and S. Nurko , “An ANMS‐NASPGHAN Consensus Document on Esophageal and Antroduodenal Manometry in Children,” Neurogastroenterology and Motility 30, no. 3 (2018): e13239, 10.1111/nmo.13239. PMC5823717 29178261

[115] D. Cassilly , S. Kantor , L. C. Knight , et al., “Gastric Emptying of a Non‐Digestible Solid: Assessment With Simultaneous SmartPill pH and Pressure Capsule, Antroduodenal Manometry, Gastric Emptying Scintigraphy,” Neurogastroenterology and Motility 20, no. 4 (2008): 311–319, 10.1111/j.1365-2982.2007.01061.x. 18194154

[116] H. O. Diaz Tartera , D. L. Webb , A. K. Al‐Saffar , et al., “Validation of SmartPill Wireless Motility Capsule for Gastrointestinal Transit Time: Intra‐Subject Variability, Software Accuracy and Comparison With Video Capsule Endoscopy,” Neurogastroenterology and Motility 29, no. 10 (2017): e13107, 10.1111/nmo.13107. 28524600

[117] B. Kuo , R. W. McCallum , K. L. Koch , et al., “Comparison of Gastric Emptying of a Nondigestible Capsule to a Radio‐Labelled Meal in Healthy and Gastroparetic Subjects,” Alimentary Pharmacology & Therapeutics 27, no. 2 (2008): 186–196, 10.1111/j.1365-2036.2007.03564.x. 17973643

[118] B. Kuo , A. A. Lee , T. Abell , et al., “The Assessment of Gastrointestinal Transit by the Atmo Capsule: A Comparison With the SmartPill Capsule,” Clinical Gastroenterology and Hepatology 23, no. 9 (2025): 1633–1641.e8, 10.1016/j.cgh.2024.12.013. 39889900

[119] W. L. Hasler , T. Abell , A. Attaluri , et al., “Accelerometric Assessment of Gastric Contractility Using the Atmo Wireless Motility Capsule,” Neurogastroenterology and Motility 38, no. 2 (2026): e70255, 10.1111/nmo.70255. 41672928

[120] G. O'Grady , C. Varghese , G. Schamberg , et al., “Principles and Clinical Methods of Body Surface Gastric Mapping: Technical Review,” Neurogastroenterology and Motility 35, no. 10 (2023): e14556, 10.1111/nmo.14556. PMC10524901 36989183

[121] A. A. Gharibans , S. Kim , D. C. Kunkel , and T. P. Coleman , “High‐Resolution Electrogastrogram: A Novel, Noninvasive Method for Determining Gastric Slow‐Wave Direction and Speed,” IEEE Transactions on Biomedical Engineering 64, no. 4 (2017): 807–815, 10.1109/TBME.2016.2579310. PMC5474202 27305668

[122] C. Varghese , N. Dachs , G. Schamberg , et al., “Expert Clinical Consensus on Body Surface Gastric Mapping Phenotypes for Gastroduodenal Disorders: ‘Auckland Classification’ v1.0,” 10.1111/nmo.70377. PMC13320621 42381563

[123] M. Camilleri , “Evaluation of Body Surface Gastric Mapping Phenotypes for Gastroduodenal Disorders: Ten Points for Discussion,” Neurogastroenterology and Motility 38, no. 8 (2026): e70427, 10.1111/nmo.70427. PMC13494052 42625273

[124] G. Gourcerol , F. Tissier , C. Melchior , et al., “Impaired Fasting Pyloric Compliance in Gastroparesis and the Therapeutic Response to Pyloric Dilatation,” Alimentary Pharmacology & Therapeutics 41, no. 4 (2015): 360–367, 10.1111/apt.13053. 25523288

[125] W. J. Snape , M. S. Lin , N. Agarwal , and R. E. Shaw , “Evaluation of the Pylorus With Concurrent Intraluminal Pressure and EndoFLIP in Patients With Nausea and Vomiting,” Neurogastroenterology and Motility 28, no. 5 (2016): 758–764, 10.1111/nmo.12772. 26813266

[126] J. Keller , G. Bassotti , J. Clarke , et al., “Advances in the Diagnosis and Classification of Gastric and Intestinal Motility Disorders,” Nature Reviews. Gastroenterology & Hepatology 15, no. 5 (2018): 291–308, 10.1038/nrgastro.2018.7. PMC6646879 29622808

[127] C. Hoad , C. Clarke , L. Marciani , M. J. Graves , and M. Corsetti , “Will MRI of Gastrointestinal Function Parallel the Clinical Success of Cine Cardiac MRI?,” British Journal of Radiology 92, no. 1093 (2019): 20180433, 10.1259/bjr.20180433. PMC6435057 30299989

[128] L. Marciani , “Assessment of Gastrointestinal Motor Functions by MRI: A Comprehensive Review,” Neurogastroenterology and Motility 23, no. 5 (2011): 399–407, 10.1111/j.1365-2982.2011.01670.x. 21276139

[129] I. M. de Zwart and A. de Roos , “MRI for the Evaluation of Gastric Physiology,” European Radiology 20, no. 11 (2010): 2609–2616, 10.1007/s00330-010-1850-3. PMC2948165 20585784

[130] M. Fox , S. Banerjee , H. Parker , and L. Marciani , “Chapter 14 – Magnetic Resonance Imaging for Gastric Motility and Function,” in Gastroparesis, ed. R. W. McCallum and H. P. Parkman (Academic Press, 2021), 175–187, 10.1016/B978-0-12-818586-5.00014-4.

[131] C. S. de Jonge , A. J. P. M. Smout , A. J. Nederveen , and J. Stoker , “Evaluation of Gastrointestinal Motility With MRI: Advances, Challenges and Opportunities,” Neurogastroenterology and Motility 30, no. 1 (2018): e13257, 10.1111/nmo.13257. 29265641

[132] W. Schwizer , H. Maecke , and M. Fried , “Measurement of Gastric Emptying by Magnetic Resonance Imaging in Humans,” Gastroenterology 103, no. 2 (1992): 369–376, 10.1016/0016-5085(92)90823-h. 1634055

[133] A. E. Bharucha , A. Manduca , D. S. Lake , et al., “Gastric Motor Disturbances in Patients With Idiopathic Rapid Gastric Emptying,” Neurogastroenterology and Motility 23, no. 7 (2011): 617, 10.1111/j.1365-2982.2011.01710.x. PMC3117921 21470342

[134] A. E. Bharucha , R. A. Karwoski , J. Fidler , et al., “Comparison of Manual and Semiautomated Techniques for Analyzing Gastric Volumes With MRI in Humans,” American Journal of Physiology – Gastrointestinal and Liver Physiology 307, no. 5 (2014): G582–G587, 10.1152/ajpgi.00048.2014. PMC4182289 25012844

[135] J. Fidler , A. E. Bharucha , M. Camilleri , et al., “Application of Magnetic Resonance Imaging to Measure Fasting and Postprandial Volumes in Humans,” Neurogastroenterology and Motility 21, no. 1 (2009): 42–51, 10.1111/j.1365-2982.2008.01194.x. PMC2676772 19019018

[136] O. Goetze , A. Steingoetter , D. Menne , et al., “The Effect of Macronutrients on Gastric Volume Responses and Gastric Emptying in Humans: A Magnetic Resonance Imaging Study,” American Journal of Physiology – Gastrointestinal and Liver Physiology 292, no. 1 (2007): G11–G17, 10.1152/ajpgi.00498.2005. 16935851

[137] C. L. Hoad , H. Parker , N. Hudders , et al., “Measurement of Gastric Meal and Secretion Volumes Using Magnetic Resonance Imaging,” Physics in Medicine and Biology 60, no. 3 (2015): 1367–1383, 10.1088/0031-9155/60/3/1367. PMC4502365 25592405

[138] P. Kunz , G. R. Crelier , W. Schwizer , et al., “Gastric Emptying and Motility: Assessment With MR Imaging‐Preliminary Observations,” Radiology 207, no. 1 (1998): 33–40, 10.1148/radiology.207.1.9530296. 9530296

[139] A. Menys , D. Keszthelyi , H. Fitzke , et al., “A Magnetic Resonance Imaging Study of Gastric Motor Function in Patients With Dyspepsia Associated With Ehlers‐Danlos Syndrome‐Hypermobility Type: A Feasibility Study,” Neurogastroenterology and Motility 29, no. 9 (2017): e13090, 10.1111/nmo.13090. 28568908

[140] D. M. Mudie , K. Murray , C. L. Hoad , et al., “Quantification of Gastrointestinal Liquid Volumes and Distribution Following a 240 mL Dose of Water in the Fasted State,” Molecular Pharmaceutics 11, no. 9 (2014): 3039–3047, 10.1021/mp500210c. 25115349

[141] J. Cho , Y. J. Lee , Y. H. Kim , et al., “Quantitative MRI Evaluation of Gastric Motility in Patients With Parkinson's Disease: Correlation of Dyspeptic Symptoms With Volumetry and Motility Indices,” PLoS One 14, no. 5 (2019): e0216396, 10.1371/journal.pone.0216396. PMC6499432 31050679

[142] T. Kanda , K. Ishii , H. Kawaguchi , K. Kitajima , and D. Takenaka , “High Signal Intensity in the Dentate Nucleus and Globus Pallidus on Unenhanced T1‐Weighted MR Images: Relationship With Increasing Cumulative Dose of a Gadolinium‐Based Contrast Material,” Radiology 270, no. 3 (2014): 834–841, 10.1148/radiol.13131669. 24475844

[143] R. D. Riordan , M. Khonsari , J. Jeffries , G. F. Maskell , and P. G. Cook , “Pineapple Juice as a Negative Oral Contrast Agent in Magnetic Resonance Cholangiopancreatography: A Preliminary Evaluation,” British Journal of Radiology 77, no. 924 (2004): 991–999, 10.1259/bjr/36674326. 15569640

[144] A. C. Silva , J. H. Lee , I. Aoki , and A. P. Koretsky , “Manganese‐Enhanced Magnetic Resonance Imaging (MEMRI): Methodological and Practical Considerations,” NMR in Biomedicine 17, no. 8 (2004): 532–543, 10.1002/nbm.945. 15617052

[145] H. Fruehauf , O. Goetze , A. Steingoetter , et al., “Intersubject and Intrasubject Variability of Gastric Volumes in Response to Isocaloric Liquid Meals in Functional Dyspepsia and Health,” Neurogastroenterology and Motility 19, no. 7 (2007): 553–561, 10.1111/j.1365-2982.2007.00904.x. 17593136

[146] J. J. L. Haans , I. M. de Zwart , P. H. C. Eilers , et al., “Gastric Volume Changes in Response to a Meal: Validation of Magnetic Resonance Imaging Versus the Barostat,” Journal of Magnetic Resonance Imaging 34, no. 3 (2011): 685–690, 10.1002/jmri.22619. 21751282

[147] I. M. de Zwart , B. Mearadji , H. J. Lamb , et al., “Gastric Motility: Comparison of Assessment With Real‐Time MR Imaging or Barostat Measurement—Initial Experience,” Radiology 224, no. 2 (2002): 592–597, 10.1148/radiol.2242011412. 12147861

[148] C. Feinle , P. Kunz , P. Boesiger , M. Fried , and W. Schwizer , “Scintigraphic validation of a magnetic resonance imaging method to study gastric emptying of a solid meal in humans,” Gut 44, no. 1 (1999): 106–111, 10.1136/gut.44.1.106. PMC1760059 9862835

[149] N. Hayakawa , Y. Nakamoto , T. F. Chen‐Yoshikawa , et al., “Gastric Motility and Emptying Assessment by Magnetic Resonance Imaging After Lung Transplantation: Correlation With Gastric Emptying Scintigraphy,” Abdom Radiology 42, no. 3 (2017): 818–824, 10.1007/s00261-016-0959-5. 27796517

[150] P. Kunz , C. Feinle , W. Schwizer , M. Fried , and P. Boesiger , “Assessment of Gastric Motor Function During the Emptying of Solid and Liquid Meals in Humans by MRI,” Journal of Magnetic Resonance Imaging 9, no. 1 (1999): 75–80, 10.1002/(sici)1522-2586(199901)9:1<75::aid-jmri10>3.0.co;2-i. 10030653

[151] A. E. Bharucha , J. L. Fidler , J. E. Huprich , et al., “A Prospective Randomized Controlled Study of Erythromycin on Gastric and Small Intestinal Distention: Implications for MR Enterography,” European Journal of Radiology 83, no. 11 (2014): 2001–2006, 10.1016/j.ejrad.2014.08.005. PMC4183713 25217123

[152] J. Borovicka , R. Lehmann , P. Kunz , et al., “Evaluation of Gastric Emptying and Motility in Diabetic Gastroparesis With Magnetic Resonance Imaging: Effects of Cisapride,” American Journal of Gastroenterology 94, no. 10 (1999): 2866–2873, 10.1016/S0002-9270(99)00448-7. 10520835

[153] S. Hosseini , R. Avci , N. Paskaranandavadivel , V. Suresh , and L. K. Cheng , “Quantification of the Regional Properties of Gastric Motility Using Dynamic Magnetic Resonance Images,” IEEE Open Journal of Engineering in Medicine and Biology 4 (2023): 38–44, 10.1109/OJEMB.2023.3261224. PMC10151011 37138590

[154] L. Neubauer , L. Forstreuter , F. Winter , et al., “Quantitative Real‐Time MRI for the Assessment of Gastric Motility,” Journal of Magnetic Resonance Imaging 63, no. 5 (2026): 1482–1495, 10.1002/jmri.70243. PMC13066535 41645687

[155] M. A. Kwiatek , A. Steingoetter , A. Pal , et al., “Quantification of Distal Antral Contractile Motility in Healthy Human Stomach With Magnetic Resonance Imaging,” Journal of Magnetic Resonance Imaging 24, no. 5 (2006): 1101–1109, 10.1002/jmri.20738. 17031837

[156] W. Ajaj , S. C. Goehde , N. Papanikolaou , et al., “Real time high resolution magnetic resonance imaging for the assessment of gastric motility disorders,” Gut 53, no. 9 (2004): 1256–1261, 10.1136/gut.2003.038588. PMC1774201 15306580

[157] K. Heissam , N. Abrehart , C. L. Hoad , et al., “Measurement of Fasted State Gastric Antral Motility Before and After a Standard Bioavailability and Bioequivalence 240 mL Drink of Water: Validation of MRI Method Against Concomitant Perfused Manometry in Healthy Participants,” PLoS One 15, no. 11 (2020): e0241441, 10.1371/journal.pone.0241441. PMC7657519 33175860

[158] H. Faas , G. S. Hebbard , C. Feinle , et al., “Pressure‐Geometry Relationship in the Antroduodenal Region in Humans,” American Journal of Physiology. Gastrointestinal and Liver Physiology 281, no. 5 (2001): G1214–G1220, 10.1152/ajpgi.2001.281.5.G1214. 11668030

[159] M. S. Hur , S. Lee , T. M. Kang , and C. S. Oh , “The Three Muscle Layers in the Pyloric Sphincter and Their Possible Function During Antropyloroduodenal Motility,” Scientific Reports 11, no. 1 (2021): 20094, 10.1038/s41598-021-99463-x. PMC8505543 34635718

[160] X. Wang , F. Alkaabi , and Z. Liu , Personalized Characterization of Gastric Dysfunctions in Gastroparesis Patients Based on MRI and Surface Mapping (DDW, 2024).

[161] X. Wang , F. Alkaabi , Y. Xia , C. Yang , and Z. Liu , Surface Modeling of Dynamic Contrast‐Enhanced MRI Enables Individualized Diagnosis of Gastric Dysmotility in Gastroparesis (ISMRM, 2025).

[162] J. Christensen , H. P. Schedl , and J. A. Clifton , “The Basic Electrical Rhythm of the Duodenum in Normal Human Subjects and in Patients With Thyroid Disease *,” Journal of Clinical Investigation 43, no. 8 (1964): 1659–1667, 10.1172/JCI105041. PMC441965 14201549

[163] S. Weston , M. Thumshirn , J. Wiste , and M. Camilleri , “Clinical and Upper Gastrointestinal Motility Features in Systemic Sclerosis and Related Disorders,” American Journal of Gastroenterology 93, no. 7 (1998): 1085–1089, 10.1111/j.1572-0241.1998.00334.x. 9672335

[164] J. D. Huizinga , J. H. Chen , Y. Fang Zhu , et al., “The Origin of Segmentation Motor Activity in the Intestine,” Nature Communications 5, no. 1 (2014): 3326, 10.1038/ncomms4326. PMC4885742 24561718

[165] Y. Kitazume , K. Takenaka , K. Ohtsuka , et al., “Motility Mapping Quantification Using the Classical Optical Flow Algorithm for Small Bowel Crohn's Disease: Comparison With Balloon‐Assisted Enteroscopy Findings,” Magnetic Resonance in Medical Sciences 22, no. 3 (2023): 325–334, 10.2463/mrms.mp.2021-0037. PMC10449560 35545505

[166] A. Menys , S. A. Taylor , A. Emmanuel , et al., “Global Small Bowel Motility: Assessment With Dynamic MR Imaging,” Radiology 269, no. 2 (2013): 443–450, 10.1148/radiol.13130151. 23801770

[167] F. Odille , A. Menys , A. Ahmed , S. Punwani , S. A. Taylor , and D. Atkinson , “Quantitative Assessment of Small Bowel Motility by Nonrigid Registration of Dynamic MR Images,” Magnetic Resonance in Medicine 68, no. 3 (2012): 783–793, 10.1002/mrm.23298. 22234983

[168] G. Chaddock , C. Lam , C. L. Hoad , et al., “Novel MRI Tests of Orocecal Transit Time and Whole Gut Transit Time: Studies in Normal Subjects,” Neurogastroenterology and Motility 26, no. 2 (2014): 205–214, 10.1111/nmo.12249. PMC4285997 24165044

[169] P. Boulby , R. Moore , P. Gowland , and R. Spiller , “Fat Delays Emptying but Increases Forward and Backward Antral Flow as Assessed by Flow‐Sensitive Magnetic Resonance Imaging,” Neurogastroenterology and Motility 11, no. 1 (1999): 27–36, 10.1046/j.1365-2982.1999.00133.x. 10087532

[170] K. S. Nayak , J. F. Nielsen , M. A. Bernstein , et al., “Cardiovascular magnetic resonance phase contrast imaging,” Journal of Cardiovascular Magnetic Resonance 17, no. 1 (2015): 71, 10.1186/s12968-015-0172-7. PMC4529988 26254979

[171] C. S. de Jonge , A. M. J. Sprengers , K. L. van Rijn , A. J. Nederveen , and J. Stoker , “Assessment of Fasted and Fed Gastrointestinal Contraction Frequencies in Healthy Subjects Using Continuously Tagged MRI,” Neurogastroenterology and Motility 32, no. 2 (2020): e13747, 10.1111/nmo.13747. PMC7027525 31828938

[172] A. M. J. Sprengers , M. P. van der Paardt , F. M. Zijta , et al., “Use of Continuously MR Tagged Imaging for Automated Motion Assessment in the Abdomen: A Feasibility Study,” Journal of Magnetic Resonance Imaging 36, no. 2 (2012): 492–497, 10.1002/jmri.23637. 22411355

[173] P. Rhee , “Analysis of Pacemaker Activity in the Human Stomach,” Journal of Physiology 589, no. 2 (2011): 6105–6118, 10.1113/jphysiol.2011.217497. PMC3286689 22005683

[174] R. Ebara , S. Ishida , T. Miyagawa , and Y. Imai , “Effects of Peristaltic Amplitude and Frequency on Gastric Emptying and Mixing: A Simulation Study,” Journal of the Royal Society Interface 20, no. 198 (2023): 20220780, 10.1098/rsif.2022.0780. PMC9810435 36596453

[175] M. Ferrua and R. Singh , “Modeling the Fluid Dynamics in a Human Stomach to Gain Insight of Food Digestion,” Journal of Food Science 75, no. 7 (2010): R151–R162, 10.1111/j.1750-3841.2010.01748.x. PMC2992692 21535567

[176] S. Kuhar , J. H. Seo , P. J. Pasricha , and R. Mittal , “In Silico Modelling of the Effect of Pyloric Intervention Procedures on Gastric Flow and Emptying in a Stomach With Gastroparesis,” Journal of the Royal Society Interface 21, no. 210 (2024): 20230567, 10.1098/rsif.2023.0567. PMC10824103 38263890

[177] A. Pal , K. Indireshkumar , W. Schwizer , B. Abrahamsson , M. Fried , and J. G. Brasseur , “Gastric Flow and Mixing Studied Using Computer Simulation,” Proceedings of the Biological Sciences 271, no. 1557 (2004): 2587–2594, 10.1098/rspb.2004.2886. PMC1691895 15615685

[178] M. Osanlouy , A. Bandrowski , B. de Bono , et al., “The SPARC DRC: Building a Resource for the Autonomic Nervous System Community,” Frontiers in Physiology 12 (2021): 12, 10.3389/fphys.2021.693735. PMC8265045 34248680

[179] E. A. Mayer , “Gut Feelings: The Emerging Biology of Gut‐Brain Communication,” Nature Reviews. Neuroscience 12, no. 8 (2011): 453–466, 10.1038/nrn3071. PMC3845678 21750565

[180] M. Camilleri , “Abnormal Gastrointestinal Motility Is a Major Factor in Explaining Symptoms and a Potential Therapeutic Target in Patients With Disorders of Gut‐Brain Interaction,” Gut 72, no. 12 (2023): 2372–2380, 10.1136/gutjnl-2023-330542. PMC10841318 37666657

[181] P. Kerlin , “Postprandial Antral Hypomotility in Patients With Idiopathic Nausea and Vomiting,” Gut 30, no. 1 (1989): 54–59, 10.1136/gut.30.1.54. PMC1378231 2920928

[182] J. Tack , P. Janssen , T. Masaoka , R. Farré , and L. Van Oudenhove , “Efficacy of Buspirone, a Fundus‐Relaxing Drug, in Patients With Functional Dyspepsia,” Clinical Gastroenterology and Hepatology 10, no. 11 (2012): 1239–1245, 10.1016/j.cgh.2012.06.036. 22813445

[183] C. Desprez , C. Melchior , F. Wuestenberghs , et al., “Pyloric Distensibility Measurement Predicts Symptomatic Response to Intrapyloric Botulinum Toxin Injection,” Gastrointestinal Endoscopy 90, no. 5 (2019): 754–760.e1, 10.1016/j.gie.2019.04.228. 31028783

[184] M. A. Khashab , S. Ngamruengphong , D. Carr‐Locke , et al., “Gastric Per‐Oral Endoscopic Myotomy for Refractory Gastroparesis: Results From the First Multicenter Study on Endoscopic Pyloromyotomy (With Video),” Gastrointestinal Endoscopy 85, no. 1 (2017): 123–128, 10.1016/j.gie.2016.06.048. 27354102

[185] J. Martinek , R. Hustak , J. Mares , et al., “Endoscopic Pyloromyotomy for the Treatment of Severe and Refractory Gastroparesis: A Pilot, Randomised, Sham‐Controlled Trial,” Gut 71, no. 11 (2022): 2170–2178, 10.1136/gutjnl-2022-326904. PMC9554080 35470243

[186] L. Yu , X. Wang , and Z. Liu , “Zero‐Shot Promptable Dynamic Gastric MRI Segmentation Based on SAM‐2,” Conf Proc IEEE Eng Med Biol Soc, 2026.

[187] N. Ravi , V. Gabeur , Y. T. Hu , et al., SAM 2: Segment Anything in Images and Videos (ICLR, 2025).

